# Rice Genotypes Express Compensatory Root Growth With Altered Root Distributions in Response to Root Cutting

**DOI:** 10.3389/fpls.2022.830577

**Published:** 2022-02-28

**Authors:** Tsubasa Kawai, Yinglong Chen, Hirokazu Takahashi, Yoshiaki Inukai, Kadambot H. M. Siddique

**Affiliations:** ^1^The UWA Institute of Agriculture, The University of Western Australia, Perth, WA, Australia; ^2^School of Agriculture and Environment, The University of Western Australia, Perth, WA, Australia; ^3^Graduate School of Bioagricultural Sciences, Nagoya University, Nagoya, Japan; ^4^International Center for Research and Education in Agriculture, Nagoya University, Nagoya, Japan

**Keywords:** root system architecture, crown root, lateral root, compensatory root growth, root cutting, rice

## Abstract

Root systems play a pivotal role in water and nutrient uptake from soil. Lateral root (LR) growth is promoted to compensate for inhibited main root growth. Compensatory LR growth contributes to maintaining total root length (TRL) and hence water and nutrient uptake in compacted soils. However, it remains unclear how shoot and root phenotypic traits change during the compensatory growth and whether there are genotypic variations in compensatory root growth. This study analyzed shoot and root morphological traits of 20 rice genotypes, which includes mutants with altered root morphology, during the vegetative stage using a semihydroponic phenotyping system. The phenotyping experiment detected large variation in root and shoot traits among the 20 genotypes. Morphological changes induced by root cutting were analyzed in six selected genotypes with contrasting root system architecture. Root cutting significantly affected root distribution along vertical sections and among diameter classes. After root cutting, more roots distributed at shallower depth and thicker LRs developed. Furthermore, genotypes with deeper root growth without root cutting allocated more compensatory roots to deeper sections even after root cutting than the genotypes with shallower rooting. Due to the compensatory LR growth, root cutting did not significantly affect TRL, root dry weight (RDW), or shoot dry weight (SDW). To analyze the interaction between crown root (CR) number and compensatory root growth, we removed half of the newly emerged CRs in two genotypes. TRL of YRL38 increased at depth with CR number manipulation (CRM) regardless of root tip excision, which was attributed to an increase in specific root length (SRL), despite no change in RDW. Taken together, the tested rice genotypes exhibited compensatory root growth by changing root distribution at depth and in diameter classes. Reducing CR number promoted root development and compensatory growth by improving the efficiency of root development [root length (RL) per resource investment].

## Introduction

Root system architecture (RSA) is the shape and distribution of a root system in soil (Rogers and Benfey, 2015). It has an important role in plant anchorage, soil water and nutrient uptake, and plant growth and yield. Rice has a fibrous root system comprising a seminal root (SR), numerous crown roots (CRs), and lateral roots (LRs) (Supplementary Figure S1A). An embryonic SR and shoot-borne CRs are important for establishing a framework to explore soil (Yamauchi et al., 1996). LRs are produced postembryonically from seminal and CRs (main roots) and play a major role in water and nutrient uptake as they occupy ∼ 90% of the TRL (Yamauchi et al., 1987). LRs are further classified into S- and L-types, according to distinct morphological and anatomical characteristics (Supplementary Figure S1B) (Kawata and Shibayama, 1965; Kono et al., 1972; Sasaki et al., 1981; Yamauchi et al., 1987). S-type LRs are short and thin and do not produce higher-order LRs, whereas L-type LRs are long and thick and often produce higher-order LRs. S-type LRs contribute to the hydraulic conductivity of the whole root system (representing water uptake ability) than L-type LRs (Watanabe et al., 2020). The production of L-type LRs is important for expanding the root system due to their ability to produce higher-order LRs. Plastic development of LRs plays an important role for the adaptation to soil water fluctuation in rice (Suralta et al., 2018; Lucob-Agustin et al., 2021).

Lateral root development is promoted when parent root growth is inhibited by soil compaction and other reasons (Bengough et al., 2006). A hard soil layer in subsoil causes mechanical impedance for parent root growth (Batey, 2009), which triggers LR development in upper loose soils in various plant species (Shierlaw and Alston, 1984; Atwell, 1990; Montagu et al., 2001; Chen et al., 2014). In field-compacted soils, the roots grow in macropores and clacks, formed by earthworms or previous root growth (White and Kirkegaard, 2010; Haling et al., 2011; Kautz et al., 2013; Han et al., 2015). Compensatory LR growth increases root exploration in these spaces, which enables roots to pass through the hard soil layer. It facilitates water and nutrient uptake by maintaining adequate TRL that contributes to shoot growth maintenance (Montagu et al., 2001).

Compensatory LR growth can be induced in various plant species using root cutting treatments (Torrey, 1950; Crossett et al., 1975; Biddington and Dearman, 1984; Sasaki et al., 1984; Van Staden and Ntingane, 1996; Vysotskaya et al., 2001; Xu et al., 2017). Root cutting would be a suitable method to analyze the ability of compensatory root growth itself without a number of chemical, physical, and biological changes brought by soil compaction (Correa et al., 2019). In rice, main root tip cutting induced L-type LR development in the remaining proximal portions and promoted elongation of first-order LRs and higher-order branching (Sasaki et al., 1984; Kawai et al., 2017). The degree of LR development after root cutting varies depending on the ratio of cut roots in an individual root (Biddington and Dearman, 1984). However, it remains unclear how much the promoted LR growth by root cutting compensates for main root growth and how it can be promoted.

Some studies have revealed that reduced CR number increases deep rooting and enhances LR proliferation. Maize genotypes with fewer CRs had deeper roots, enhancing nitrogen (N) acquisition under low N and increasing biomass (Saengwilai et al., 2014). This deeper rooting increases access to subsoil water, which improves drought tolerance (Gao and Lynch, 2016). Reduced CR number through CR removal enhanced deeper rooting and relocation of biomass to LRs, which improves shoot growth in maize under low N (Guo and York, 2019). These studies suggest that reduced CR number could improve plant resilience to environmental stresses by improving RSA. However, the relationship between CR number and compensatory root growth remains unclear.

A semihydroponic phenotyping system was developed to characterize root trait variability in food crops (Chen et al., 2011), which includes narrow-leafed lupin (*Lupinus angustifolius* L.) (Chen et al., 2011, 2012, 2016), chickpea (*Cicer arietinum* L.) (Chen et al., 2017), maize (*Zea mays* L.) (Qiao et al., 2019), wheat (*Triticum aestivum* L.) (Chen et al., 2020), barley (Wang et al., 2021), and soybean (Liu et al., 2021). The semihydroponic system is advanced, as root growth can be easily monitored and manipulated. A study has tested the feasibility of using this system for observing dynamic root growth following root cutting in narrow-leafed lupin (Chen et al., unpublished data). It would be an efficient system for studying root trait variability in rice genotypes, which includes root responses to root tip cutting and CR number manipulation (CRM).

Thus, this study used the semihydroponic phenotyping system to characterize variation in shoot and root morphological traits in 20 rice genotypes, which includes Australian and Japanese genotypes and mutants with altered root phenotypes (Experiment I). Six genotypes with contrasting root systems, selected from Experiment I, were examined for compensatory root growth following root cutting on the main roots (Experiment II). In addition, we reduced CR number by removing newly emerged CRs in two selected genotypes (Experiment III) to reveal the relationships between CR number and the degree of compensatory growth.

## Materials and Methods

### Phenotyping (Experiment I)

#### Experimental Design, Setup, and Plant Material

**FIGURE 1:**
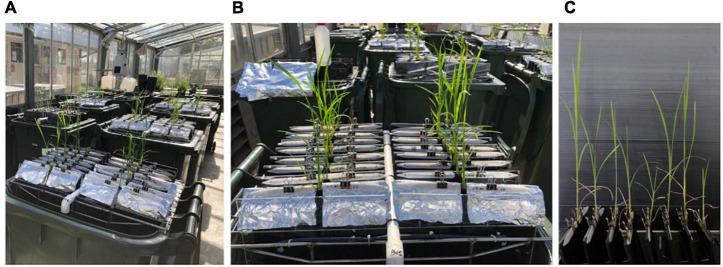
**(A)** Rice seedlings grown in a semihydroponic phenotyping platform. **(B)** Close up of plants in the bin. **(C)** Shoots from the side.

Experiment I used 20 genotypes of lowland rice (*Oryza sativa* L.), which includes ten Australian genotypes, five Japanese genotypes, and five rice mutants with altered root phenotypes (parent Taichung 65) (Table 1). The semihydroponic phenotyping system was used for all three experiments and set up according to Chen et al. (2011; Figure 1). Briefly, the system comprises 240 L plastic wheelie bin (108 cm height × 75 cm length × 58 cm width), 16 growth units, and an automatically controlled irrigation system. The growth units are made of a 5-mm thick acrylic panel (260 mm × 480 mm) wrapped in black calico cloth. Sixteen plants were grown per bin with five or six replications per genotype. Genotypes were randomly allocated in seven bins with only one plant per genotype grown in a bin. At harvest, three to six replications per genotype were analyzed their shoot and root traits.

**TABLE 1:** Rice genotypes used in this study.

Australian genotypes	Bogan, Calrose (Originated from US), Goolarah, Inga, Langi, Pelde, Quest, Reiziq, YRL38, YRW4	
Japanese genotypes	Akihikari, Koshihikari, Nipponbare, Somewake, Taichung 65	

Mutants	*crl1*	Reduced crown roots (Inukai et al., 2005)
	T12-3, T12-36	Increased L-type LRs and decreased S-type LRs and main root growth (Kawai et al., under review)
	*Osiaa13*	Reduced LRs and gravitropism (Kitomi et al., 2012)
	*qhb/Oswox5*	Reduced CRs and S-type LRs and increased L-type LRs after root cutting (Kawai et al., 2022)

The bins were filled with 30 L of a complete nutrient solution previously used for hydroponic rice cultivation (Colmer, 2003; Supplementary Table S1) and refreshed weekly. Rice plants were irrigated continuously with a pomp so that the plants were not water stressed. The amount of evaporated water from the blank bin without plants was measured every 2 days, with the same amount of distilled water added to the bins with plants to maintain the water level and concentration of the nutrient solution. Plants were grown in a naturally lit glasshouse at The University of Western Australia (31°93′ S, 115°83′ E) with day–night temperatures of 30–20°C and photoperiod of 10–14 h from late April to mid June 2020 (Experiment I) and from late October to early December 2020 (Experiments II and III).

#### Seed Germination

Rice seeds were soaked in tap water at 30–20°C for 3 days and then transferred onto plastic nets floating on 10% strength nutrient solution (Supplementary Table S1) to grow in the same glasshouse as above for 3 days. Uniform germinated seeds with approximately 1 cm of coleoptile and 2–3 cm of SR were transplanted carefully into the growth units of the semihydroponic system. One individual plant was grown in each growth unit with 16 growth units installed per bin (seven bins in total). A blank bin without plants was used to measure the amount of evaporated water from each bin during the experiment.

### Root Cutting Treatment (Experiment II)

Root cutting treatment was conducted using six rice genotypes (Nipponbare, Taichung 65, Bogan, YRL38, *crl1*, and *qhb/Oswox5*) selected from Experiment I. The semihydroponic system was used with the same settings as Experiment I. In Experiments II and III, sixteen plants were grown per bin with six replications per genotype–treatment (five bins in total). Genotypes–treatments were randomly allocated in each bin with the same set of genotypes–treatments in each bin. At harvest, three to six replications per genotype–treatment were analyzed for their shoot and root traits. Root growth of each plant was monitored and photographed every 5–6 days by opening the wrapped cloth from the back of each growth unit after lifting it out of the bin and placing it on a flat bench. The root cutting treatment started 6 days after transplanting (DAT) when the first main root (SR) reached the cutting depth (7.5–12.5 cm below stem base) in most plants. Main roots that reached the depth were cut 5 mm behind the root tip (Supplementary Figure S2A). Plants without root cutting were grown as the control (Cont). The root cutting treatment was conducted at 6, 11, 16, 21, 27, and 32 DAT. Shoot height (SH), leaf number (LN), tiller number (TN), and maximum root depth (MRD) were measured.

### Crown Root Number Manipulation (Experiment III)

The semihydroponic system was used, with the same settings as Experiments I and II. Two genotypes Taichung 65 and YRL38 were selected from Experiment I for CRM by removing 50% of newly emerged CRs from the basal part (Supplementary Figure S2B). Shorter CRs among the newly emerged CRs were removed. The CRM was conducted on the same day of root cutting treatment in both genotypes with and without the root cutting treatment, as explained above.

### Sampling and Measurements

Plants were assessed at 55 DAT in Experiment I and 41DAT in Experiments II and III. The growth panel in each bin was removed and placed on a flat bed. Plants were photographed with a digital camera after opening the wrapped cloth of each growth panel. SH and TN were measured, and then, shoots were cut from the stem base. MRD and seminal and crown root number (SCRN) were measured manually. The leaves were scanned at 200 dpi with a desktop scanner (Epson Perfection V800; Epson, CA, United States) for leaf area measurement using WinRHIZO Pro software (v2009; Regent Instruments, Montreal, QC, Canada). The leaves and stems were combined and oven-dried at 60°C for 48 h to determine shoot dry weight (SDW). Roots were sampled in four sections below the stem base (section 1, 0–10 cm, s1; section 2, 10–20 cm, s2; section 3, 20–30 cm, s3; section 4, >30 cm, s4). Root samples were separated to avoid crossover and scanned at 600 dpi with a desktop scanner. TRL and root diameter (RD) length [root length (RL) in five diameter classes (<80 μm, S-type LRs, RL_S; 80–150 μm, S- and L-type LRs, RL_M; 150–300 μm, L-type LRs, RL_L; 300–500 μm, thick L-type LRs and thin CRs, RL_LL; >500 μm, seminal and CRs, RL_CR)] were recorded by analyzing root images using WinRHIZO Pro software. The main RD was estimated from RL in diameter classes 500–2000 μm. After scanning, root subsamples for the same plant were combined to determine root dry weight (RDW) per plant. Detailed descriptions of the 32 root traits and five shoot-related traits are listed in Table 2.

**TABLE 2:** Description of 32 root-related traits and five shoot traits in rice characterized in a semihydroponic phenotyping system.

Trait	Abbreviation	Description	Unit
**Major traits**			
Maximum root depth	MRD	The longest root length (seminal or crown root)	cm
Seminal and crown root number	SCRN	Seminal and crown root number	number per plant
Total root length	TRL	Total root length	cm
Root diameter	RD	Average root diameter	μm
Root area	RA	Root surface area	cm^2^
Root volume	RV	Root volume	cm^3^
Specific root length	SRL	Total root length per unit root dry mass	cm mg^–1^
Root length intensity	RLI	Total root length per unit root depth	cm cm^–1^
Root tissue density	RTD	Root dry mass per unit root volume	mg cm^–3^
Root dry weight	RDW	Root dry mass	mg
Shoot dry weight	SDW	Shoot dry mass	mg
Total dry weight	TDW	Total dry mass (sum of root and shoot dry mass)	mg
Shoot to root ratio	SRR	Shoot-to-root dry mass ratio	mg mg^–1^
Shoot height	SH	Shoot height measured to the tallest leaf	cm
Leaf number	LN	Leaf number of main stem	Number per plant
Leaf area	LA	Leaf area	cm^2^
Tiller number	TN	Tiller number	Number per plant
**Local traits**			
Root length s1	RL_s1	Root length in section 1 (s1, 0–10 cm; above cutting depth)	cm
Root length s2	RL_s2	Root length in section 2 (s2, 10–20 cm)	cm
Root length s3	RL_s3	Root length in section 3 (s3, 20–30 cm)	cm
Root length s4	RL_s4	Root length in section 4 (s4, >30 cm)	cm
Root length s2–4	RL_s2–4	Root length in section 2–4 (s2–4, >10 cm; below cutting depth)	cm
Root diameter length S	RL_S	Root length with diameter < 80 μm (S-type lateral root)	cm
Root diameter length M	RL_M	Root length with diameter 80–150 μm (S- and L-type lateral root)	cm
Root diameter length L	RL_L	Root length with diameter 150–300 μm (L-type lateral root)	cm
Root diameter length LL	RL_LL	Root length with diameter 300–500 μm (thick L-type lateral root and thin crown root)	cm
Root diameter length CR	RL_CR	Root length with diameter > 500 μm (seminal and crown root)	cm
Root length proportion s1	RLP_s1	Proportion of RL_s1 per total root length	%
Root length proportion s2	RLP_s2	Proportion of RL_s2 per total root length	%
Root length proportion s3	RLP_s3	Proportion of RL_s3 per total root length	%
Root length proportion s4	RLP_s4	Proportion of RL_s4 per total root length	%
Root length proportion s2–4	RLP_s2–4	Proportion of RL_s2–4 per total root length	%
Root length proportion S	RLP_S	Proportion of RL_S per total root length	%
Root length proportion M	RLP_M	Proportion of RL_M per total root length	%
Root length proportion L	RLP_L	Proportion of RL_L per total root length	%
Root length proportion LL	RLP_LL	Proportion of RL_LL per total root length	%
Root length proportion CR	RLP_CR	Proportion of RL_CR per total root length	%

To evaluate the degree of compensatory root growth, compensation rate was calculated as follows: (1) the compensatory RL was calculated for each genotype as in Equation 1. If root growth does not respond to root cutting at all, compensatory RL will be zero, and (2) the compensation rate for each genotype was calculated as in Equation 2. Compensation rate indicates how much RL below the cutting depth in the control was compensated by increased RL above the depth and/or LR growth below the depth after root cutting, and (3) the proportion of compensatory RL in each section to the total was calculated as in Equation 3. For section 1, the increase in RL by root cutting than the control was defined as compensatory RL of this section.

Equation 1 (compensatory RL):


$$T\,o\,t\,a\,l\,r\,o\,o\,t\,l\,e\,n\,g\,t\,h_{C\,u\,t\,t\,r\,e\,a\,t\,m\,e\,n\,t}\,\left(c\,m\right)$$

$$-R\,o\,o\,t\,l\,e\,n\,g\,t\,h\,a\,b\,o\,v\,e\,t\,h\,e\,c\,u\,t\,d\,e\,p\,t\,h_{C\,o\,n\,t}\,\left(c\,m\right)$$

Equation 2 (Compensation rate):


$$\frac{C\,o\,m\,p\,e\,n\,s\,a\,t\,o\,r\,y\,r\,o\,o\,t\,l\,e\,n\,g\,t\,h\,\left(c\,m\right)}{R\,o\,o\,t\,l\,e\,n\,g\,t\,h\,b\,e\,l\,o\,w\,t\,h\,e\,c\,u\,t\,d\,e\,p\,t\,h_{C\,o\,n\,t}\,\left(c\,m\right)}\times 100$$

Equation 3 (Proportion of compensatory RL in each section):


R⁢o⁢o⁢t⁢l⁢e⁢n⁢g⁢t⁢h      R⁢o⁢o⁢t⁢l⁢e⁢n⁢g⁢t⁢h

$$\frac{i\,n\,s\,1_{C\,u\,t\,t\,r\,e\,a\,t\,m\,e\,n\,t}\,\left(c\,m\right)-i\,n\,s\,1_{C\,o\,n\,t}\,\left(c\,m\right)}{C\,o\,m\,p\,e\,n\,s\,a\,t\,o\,r\,y\,r\,o\,o\,t\,l\,e\,n\,g\,t\,h\,\left(c\,m\right)}\times 100$$

(for section 1, above the root cutting depth)


$$\frac{R\,o\,o\,t\,l\,e\,n\,g\,t\,h\,i\,n\,a\,s\,e\,c\,t\,i\,o\,n_{C\,u\,t\,t\,r\,e\,a\,t\,m\,e\,n\,t}\,\left(c\,m\right)}{C\,o\,m\,p\,e\,n\,s\,a\,t\,o\,r\,y\,r\,o\,o\,t\,l\,e\,n\,g\,t\,h\,\left(c\,m\right)}\times 100$$

(for sections 2–4, below the root cutting depth)

### Statistical Analysis

Differences in morphological traits among genotypes (with and without root cutting, and with and without CRM) were compared using one (two or three)-way ANOVA and a multiple-comparison Tukey’s test using the glht function from multcomp package version 1.4-13 (Hothorn et al., 2008) in Rstudio version 1.2.5033 (R Core Team, 2020). Differences between with and without root cutting were compared using a two-tailed Student’s *t*-test. Pearson’s correlation analysis was performed using corrplot package version 0.84 (Wei and Simko, 2017) in Rstudio. The major traits without root area (RA) and root volume (RV) were used for principal component analysis (PCA) using FactoMineR package version 2.3 (Lê et al., 2008) in Rstudio.

## Results

### Phenotypic Variations Among Rice Genotypes

At 55 DAT, significant differences (*p* < 0.01) among the 20 genotypes were observed in most of the measured shoot and root traits except root tissue density (RTD) (Table 3). Among the 15 major traits, the highest CV was detected in TRL (0.51), followed by RA (0.47), RV (0.44), and RDW (0.42) (Table 3).

**TABLE 3:** Descriptive statistics of 15 major traits in 20 rice genotypes grown in a semihydroponic phenotyping system for 55 days after transplanting.

Trait	Abbreviation	Minimum	Maximum	Mean	Median	SD	CV	Significance
Maximum root depth (cm)	MRD	16.5	43.9	29.9	29.2	6.6	0.22	***
Seminal and crown root number	SCRN	1.8	28.3	17.1	17.8	5.6	0.33	***
Total root length (cm)	TRL	416	2782	1302	1203	668	0.51	***
Root diameter (μm)	RD	165	283	210	208	30	0.14	***
Root area (cm^2^)	RA	34.0	179.1	80.7	77.2	37.7	0.47	***
Root volume (cm^3^)	RV	0.17	0.96	0.42	0.40	0.18	0.44	***
Specific root length (cm mg^–1^)	SRL	14.2	43.2	26.3	27.6	6.4	0.24	***
Root length intensity (cm cm^–1^)	RLI	12.5	73.5	41.1	41.8	14.3	0.35	**
Root tissue density (mg cm^–3^)	RTD	100	162	121	115	16	0.14	n.s.
Root dry weight (mg)	RDW	22	107	49	45	20	0.42	***
Shoot dry weight (mg)	SDW	93	299	176	152	65	0.37	***
Total dry weight (mg)	TDW	115	363	225	197	80	0.36	***
Shoot to root ratio (mg mg^–1^)	SRR	1.9	5.9	4.0	4.0	1.0	0.25	***
Shoot height (cm)	SH	22.2	41.2	31.9	31.6	5.4	0.17	**
Leaf number	LN	4.8	6.3	5.4	5.3	0.5	0.09	***

Significance was based on one-way ANOVA of the 20 genotypes (n = 3–6) (**P < 0.01; ***P < 0.001; n.s., not significant).

Pearson’s correlation coefficients were calculated for the major traits (Supplementary Table S2). All traits, except RTD, had strong correlations (*p* < 0.01) with at least one of the other traits. Significant correlations were detected among root traits (Supplementary Table S2). TRL positively correlated with MRD (Supplementary Figure S3A), RA, RV, specific root length (SRL; Supplementary Figure S3B), root length intensity (RLI; Supplementary Figure S3C), and RDW (Supplementary Figure S3D and Supplementary Table S2). In contrast, TRL negatively correlated with RD (Supplementary Figure S3E). RD also negatively correlated with MRD, SRL (Supplementary Figure S3F), and RLI (Supplementary Table S2). SCRN positively correlated with RV and RDW (Supplementary Figure S3G) but negatively correlated with SRL (Supplementary Figure S3H and Supplementary Table S2). SRL also positively correlated with MRD and RLI (Supplementary Figure S3I and Supplementary Table S2). RDW positively correlated with MRD, RA, RV, and RLI (Supplementary Table S2). All root traits were highly correlated with at least one shoot trait (mostly *p* < 0.01), except for SCRN and RTD (Supplementary Table S2). TRL and RDW positively correlated with SDW, SH, and LN (Supplementary Table S2). A negative correlation occurred between RD and SH (Supplementary Table S2). Positive correlations occurred within shoot traits, except between LN and SH (Supplementary Table S2). The shoot to root ratio (SRR) negatively correlated with SCRN (Supplementary Figure S3J), RV, and RDW (Supplementary Table S2).

**FIGURE 2:**
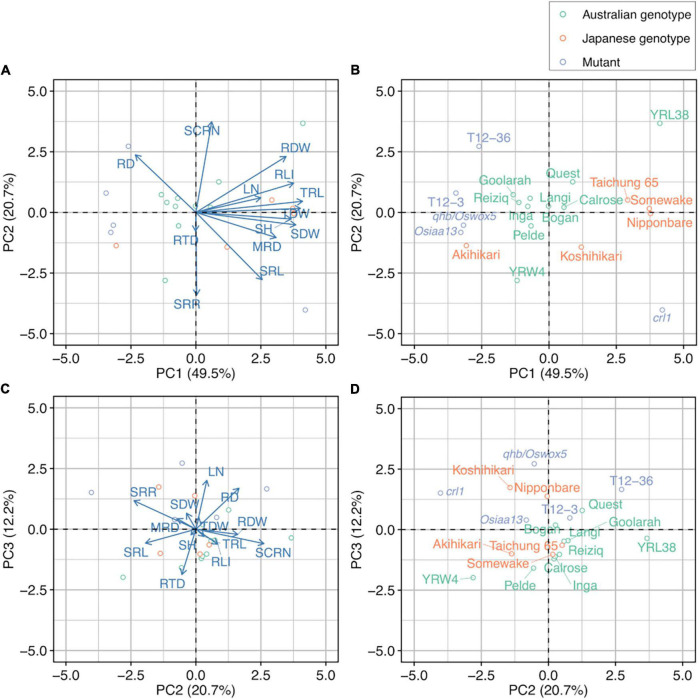
Principal component analysis of 13 selected major traits in 20 rice genotypes grown in a semihydroponic phenotyping system for 55 days after transplanting. Biplot for **(A)** PC1 and PC2 and **(C)** PC2 and PC3. Corresponding distribution of 20 rice genotypes for **(B)** PC1 and PC2 and **(D)** PC2 and PC3.

Principal component analysis was performed for 13 selected major traits (excluding mathematically linked traits, RA and RV) to determine phenotypic variation (Table 4). Four principal components with eigenvalues > 1 were detected, which explains 91.1% of the genotypic variability (Table 4). PC1 accounted for 49.5% of the total variability and positively correlated with MRD, TRL, RLI, RDW, SDW, total dry weight, and SH (Table 4 and Figure 2A). PC2 accounted for 20.7% of the total variability and was positively correlated with SCRN and negatively correlated with SRL and SRR (Table 4 and Figures 2A,C). PC3 accounted for 12.2% of the total variability and positively correlated with RD and LN (Table 4 and Figure 2C). The biplots showed a separation of rice genotypes from Japan and Australia and rice mutants, except for Akihikari (Japanese genotype), YRL38 (Australian genotype), and *crl1* along PC1 axis (Figures 2A,B) but not PC2 axis (Figures 2C,D).

**TABLE 4:** Loading scores of 13 selected traits and eigenvalues and the proportion of each principal component variance.

Trait	PC1	PC2	PC3	PC4
MRD	**0.73**	–0.24	0.15	–0.29
SCRN	0.14	**0.88**	–0.20	0.19
TRL	**0.97**	0.11	–0.10	–0.12
RD	–0.55	0.56	**0.57**	0.07
SRL	0.60	**–0.65**	–0.19	–0.35
RLI	**0.89**	0.28	–0.21	–0.09
RTD	0.00	–0.18	–0.63	**0.72**
RDW	**0.82**	0.54	–0.07	–0.02
SDW	**0.91**	–0.12	0.22	0.28
TDW	**0.95**	0.04	0.16	0.22
SRR	0.01	**–0.81**	0.40	0.40
PH	**0.87**	–0.06	–0.08	0.06
LN	0.59	0.14	**0.68**	0.21
Variation proportion				
Eigenvalue	6.4	2.7	1.6	1.1
Variance (%)	49.5	20.7	12.2	8.7
Cumulative variance (%)	49.5	70.2	82.4	91.1

*For each trait, the largest loading score (absolute value) among the four components are in bold.*

### Effect of Root Cutting Treatment on Shoot and Root Growth

#### Results of Root Cutting Treatment

Based on the phenotyping results, we selected six genotypes for their contrasting root phenotypes. In the PCA, Nipponbare, Taichung 65, YRL38, and *crl1* mutant had similar PC1 scores but dispersed along the PC2 axis (Figure 2B). Bogan and the *qhb/Oswox5* mutant had similar PC2 scores with Nipponbare and Taichung 65 but smaller PC1 scores than these genotypes (Figure 2B). The first main root (SR) reached the cutting depth (7.5–12.5 cm below stem base) in most plants at 6 DAT, when the root cutting treatment started. The cut root number ranged from 1 in *crl1* to 14.3 in Nipponbare (Supplementary Figure S4A). The cumulative number of cut roots increased linearly with time in most genotypes except *crl1* (Supplementary Figure S4B). In the *crl1* mutant, only one main root (SR) was cut at 6 DAT; no CRs had reached the cutting depth by 32 DAT. The average root cutting depth was around 10 cm and did not differ among genotypes (Supplementary Figure S4C).

#### Effect of Root Cutting Treatment on Shoot and Root Growth

**FIGURE 3:**
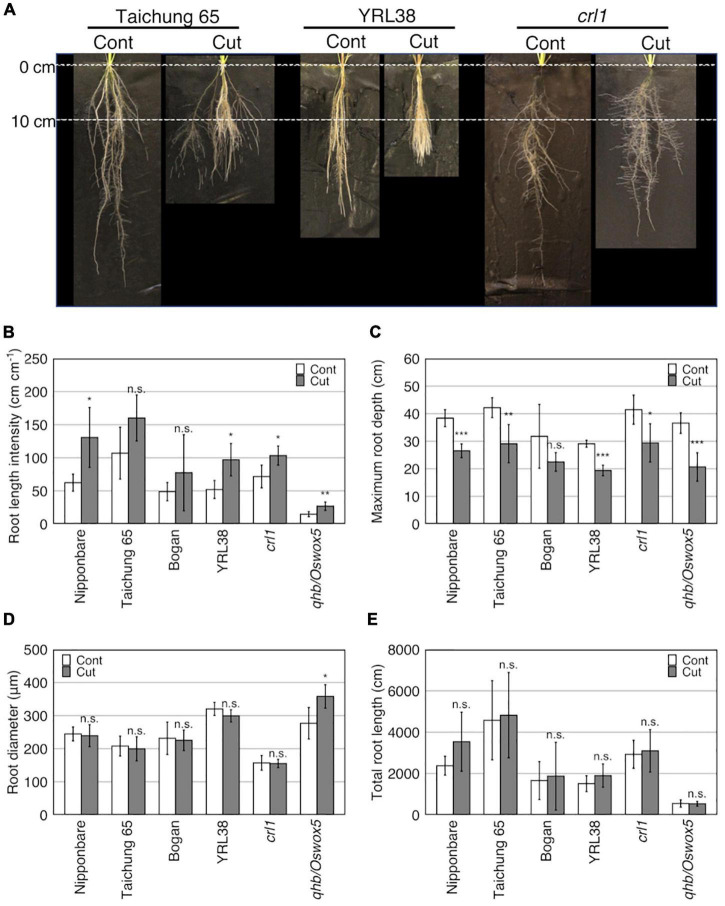
Effect of root cutting treatment on root traits in six rice genotypes grown in a semihydroponic phenotyping platform for 41 days after transplanting (DAT). **(A)** Root phenotypes of three rice genotypes (Taichung 65, YRL38, and *crt1*) at 41DAT with cut (Cut) and without cut (Cont). **(B–E)** Effects of root cutting treatment on root traits. Values represent mean ± SD (*n* = 3–6) (*p < 0.05; **p < 0.01; ***p < 0.001; n.s., not significant).

At 41 DAT, most measured traits varied among genotypes (*p* < 0.05), except for RTD and LN (Table 5 and Supplementary Table S3). Only two root traits, MRD and RLI, significantly differed between the control and root cutting treatment (*p* < 0.001) (Table 5 and Supplementary Table S3). The root cutting treatment decreased MRD but increased RLI in most genotypes (Figures 3A–C) and RD in the *qhb/Oswox5* mutant (Figure 3D). A genotype × treatment interaction was detected for RD (*p* < 0.05) (Table 5). TRL differed among genotypes (*p* < 0.001) (Table 5 and Supplementary Table S3), but no significant differences occurred between the control and root cutting treatment in each genotype (Figure 3E). These data indicate that root cutting changed the root distribution among vertical sections.

**TABLE 5:** Descriptive statistics of 17 shoot and root traits (referred as major traits) in six rice genotypes with and without root cutting treatment grown in a semihydroponic phenotyping platform for 41 days after transplanting.

Trait	Abbreviation	Minimum	Maximum	Mean	Median	SD	CV	Significance		
								G	T	G × T
Maximum root depth (cm)	MRD	19.4	42.2	30.6	29.3	7.8	0.25	***	***	n.s.
Seminal and crown root number	SCRN	1.0	32.5	18.6	22.0	9.8	0.53	***	n.s.	n.s.
Total root length (cm)	TRL	538	4820	2446	2137	1395	0.57	***	n.s.	n.s.
Root diameter (μm)	RD	155	358	243	235	62	0.26	***	n.s.	*
Root area (cm^2^)	RA	49.3	288.2	165.3	149.8	78.6	0.48	***	n.s.	n.s.
Root volume (cm^3^)	RV	0.37	1.51	0.96	0.93	0.43	0.44	***	n.s.	n.s.
Specific root length (cm mg^–1^)	SRL	10.4	59.6	27.5	24.6	16.1	0.58	***	n.s.	n.s.
Root length intensity (cm cm^–1^)	RLI	14.8	160.2	79.3	74.4	42.4	0.53	***	***	n.s.
Root tissue density (mg cm^–3^)	RTD	89.4	112.3	99.4	97.4	7.1	0.07	n.s.	n.s.	n.s.
Root dry weight (mg)	RDW	35	163	95	93	46	0.48	***	n.s.	n.s.
Shoot dry weight (mg)	SDW	114	358	236	239	94	0.40	***	n.s.	n.s.
Total dry weight (mg)	TDW	149	521	331	364	125	0.38	***	n.s.	n.s.
Shoot to root ratio (mg mg^–1^)	SRR	1.7	6.6	3.0	2.1	1.8	0.60	***	n.s.	n.s.
Shoot height (cm)	SH	21.5	40.6	29.6	29.3	7.0	0.23	***	n.s.	n.s.
Leaf number	LN	5.0	6.0	5.6	5.5	0.3	0.06	n.s.	n.s.	n.s.
Leaf area (cm^2^)	LA	9.2	32.8	20.6	21.3	8.0	0.39	***	n.s.	n.s.
Tiller number	TN	0.3	2.3	1.2	1.3	0.5	0.42	*	n.s.	n.s.

Significance was based on two-way ANOVA (n = 3–6) (*p < 0.05; ***p < 0.001; n.s., not significant). G, genotype; T, treatment.

Significant differences for the four measured traits (MRD, SH, LN, and TN) were detected among genotypes for at least one time point (*p* < 0.05) (Supplementary Table S4). The MRD also significantly differed between the control and root cutting treatment after 11 DAT (*p* < 0.001) (Supplementary Table S4). In most genotypes, MRD increased linearly with DAT in the control but did not increase for the first 10 days after the first root cutting, which later increases but not reaching the control values (Supplementary Figure S5).

#### Effect of Root Cutting Treatment on Root Distribution

**FIGURE 4:**
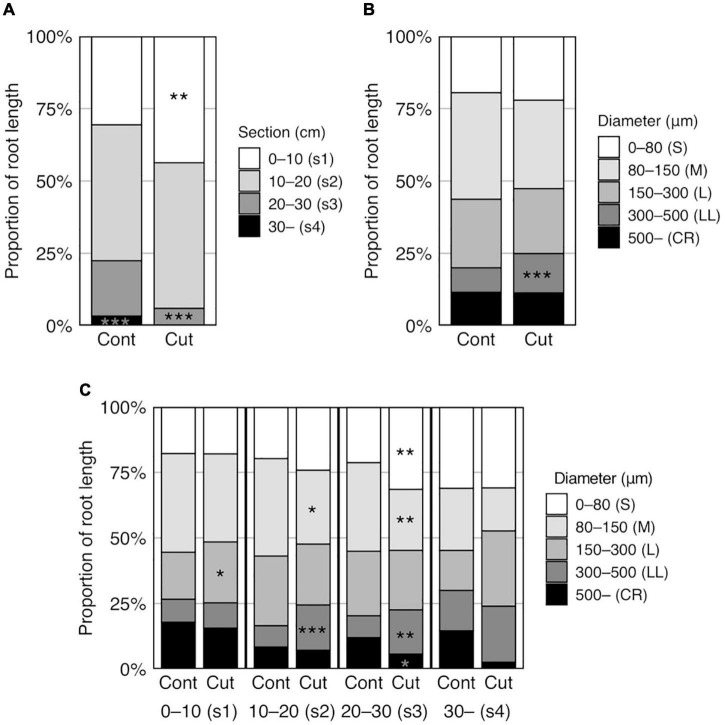
Effect of root cutting treatment on root distribution in six rice genotypes grown in a semihydroponic phenotyping platform for 41 days after transplanting. **(A)** Proportion of RL in four sections. **(B)** Proportion of RL in five diameter classes. **(C)** Proportion of RL in five diameter classes in each section. Values represent means (*n* = 28 and 23, Cont and Cut, respectively) (*p < 0.05; **p < 0.01; ***p < 0.001).

**FIGURE 5:**
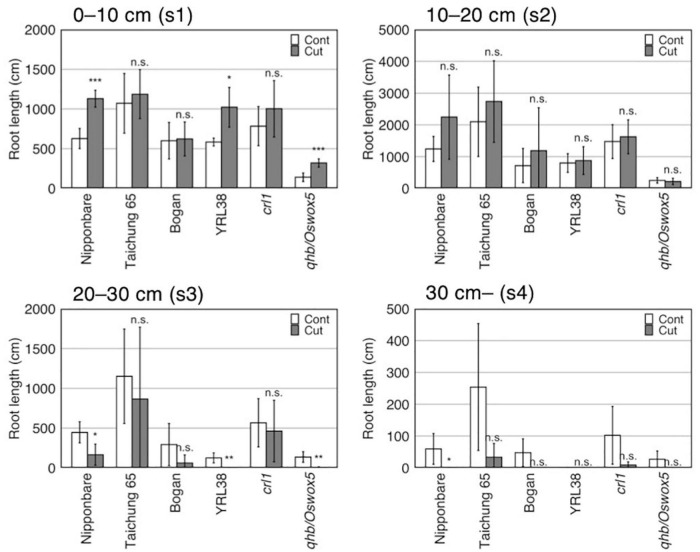
Effect of root cutting treatment on RL in four sections in six rice genotypes grown in a semihydroponic phenotyping platform for 41 days after transplanting. Values represent mean ± SD (*n* = 3–6) (*p < 0.05; **p < 0.01; ***p < 0.001; n.s., not significant).

Root cutting affected the RLs in some sections (*p* < 0.01) (Supplementary Table S5), which increases in section 1 (0–10 cm) and decreasing in section 4 (> 30 cm) (Supplementary Table S6), although TRL did not differ between the control and root cutting treatment (Figure 3E, Table 5, and Supplementary Table S3). The section RL proportion (of total root system) increased in section 1 and decreased in sections 3 (20–30 cm), 4 (>30 cm), and 2–4 (>10 cm) (Figure 4A and Supplementary Table S6). Among genotypes, root cutting significantly increased RL in section 1 (RL_s1) in Nipponbare, YRL38, and *qhb/Oswox5* and decreased RL_s3 in Nipponbare, YRL38, and *qhb/Oswox5*, and RL_s4 in Nipponbare (Figure 5).

Root distribution in diameter length also changed with root cutting (*p* < 0.001) (Supplementary Table S5). For RD classes > 300 μm (Supplementary Figure S6A), RD length of 300–500 μm (RL_LL, Large L-type LRs) and its proportion of the total root system (RLP_LL) increased with root cutting (Figure 4B and Supplementary Table S6). Among root sections, RLP_LL increased in sections 2 and 3 with root cutting (Figure 4C). In contrast, the proportion of RD length of 80–150 μm (RLP_M) decreased in sections 2 and 3 (Figure 4C). In addition, the proportion of RD length of <80 μm (RLP_S) increased and >300 μm (RLP_CR) decreased with root cutting in section 3 (Figure 4C). Genotype × treatment interactions occurred for some diameter classes (Supplementary Table S5). RL_M decreased in *qhb/Oswox5*, whereas RL_LL increased in Nipponbare, Taichung 65, and YRL38 with root cutting (Supplementary Figure S6B).

The effect of root cutting on RD length in each section was analyzed for each genotype (Supplementary Figure S7). In section 1, Nipponbare and YRL38 increased RL in most diameter classes except RL_CR with root cutting. *qhb/Oswox5* increased RL in diameter classes > 150 μm. RL_LL also increased in Taichung 65. In section 2, RL_LL increased in Nipponbare, Taichung65, and YRL38. In section 3, RL_M and RL_CR decreased in Nipponbare, and RLs in all classes decreased in YRL38 and *qhb/Oswox5*. In section 4, all genotypes decreased RD length in all classes, with significant decreases detected in RL_CR in Nipponbare and RL_S, RL_LL, and RL_CR in Taichung 65.

#### Distribution of Compensatory Roots in Vertical Sections

**FIGURE 6:**
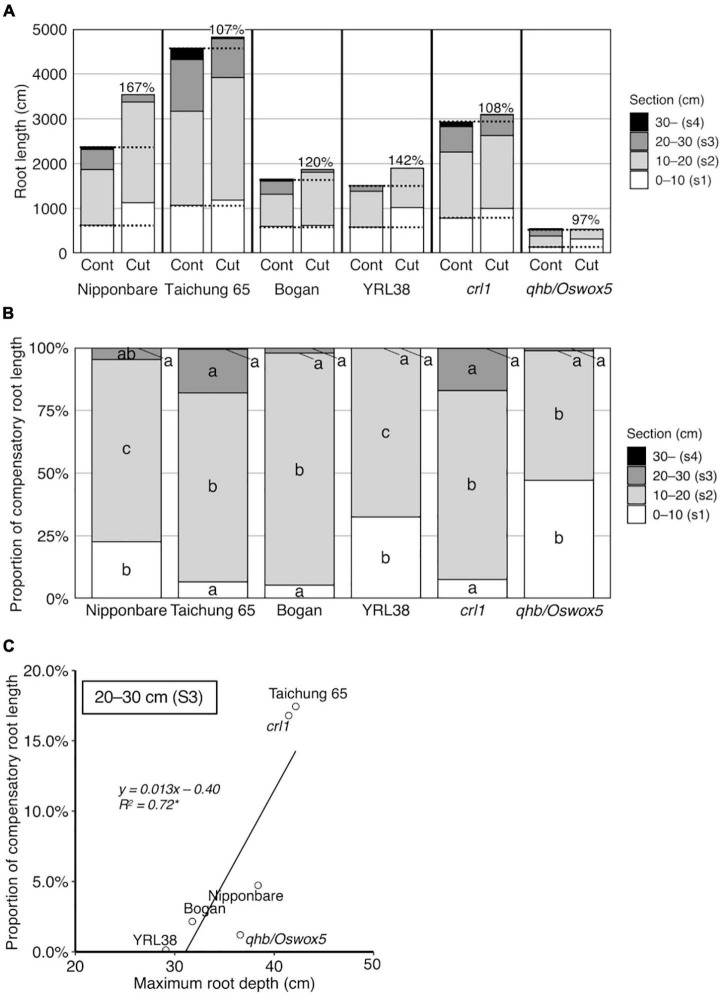
Compensatory root growth in response to the root cutting treatment in six rice genotypes grown in a semihydroponic phenotyping platform for 41 days after transplanting. **(A)** RL in four sections. Percentages on bars indicate the compensation rate for each genotype. **(B)** Proportion of compensatory RL in four sections. Values represent means (*n* = 3–6). Bar data with different letters indicate significant differences among sections in each genotype (*p* < 0.05). **(C)** Correlations between MRD in the control and proportion of compensatory RL in section 3 (*p < 0.05).

The compensation rate was calculated for each genotype to evaluate the degree of compensatory root growth after root cutting. The calculated compensation rates ranged from 97% in *qhb/Oswox5* to 167% in Nipponbare (Figure 6A, no significant difference among genotypes at α = 0.05), which indicates that root growth recovered well in all genotypes after root cutting. The proportion of RL in each section showed the sectional root distribution contributing to the compensatory RL. It was highest in section 2 for all genotypes and >50% of compensatory RL (Figure 6B). Nipponbare, YRL38, and *qhb/Oswox5* had relatively higher proportions of compensatory RL in section 1 than sections 3 and 4 (Figure 6B). In Taichung 65 and *crl1*, compensatory RL in sections 1, 3, and 4 equally contributed to the total compensatory RL (Figure 6B). These data indicate that the allocation of compensatory RL to sections differed among genotypes.

The correlation analysis revealed root distribution relationships at depth between the control and root cutting treatment, with positive correlations for MRD (Supplementary Figure S8A) and RL in each section (Supplementary Table S7). Among root sections, the proportion of compensatory RL in section 3 positively correlated with MRD in the control (Figure 6C and Supplementary Figure S8B). These data indicate that genotypes with deeper roots in the control allocated more roots to deeper sections even after root cutting.

### Effect of Crown Root Number Manipulation on Shoot and Root Growth

#### Results of Crown Root Number Manipulation

Crown root number was reduced by removing 50% of newly emerged CRs (CRM) in Taichung 65 and YRL38 to analyze the relationships between CR number and the degree of compensatory root growth. These two genotypes were selected for their contrasting root phenotypes based on the PCA in the phenotyping experiment, which includes SCRN, SRL, and SRR (PC2) with similar plant size (PC1) (Figures 2A,B). The CRM reduced CR numbers during early growth in both genotypes (Supplementary Figure S9A), whereas CR numbers did not differ with and without CRM during later growth, except for Taichung 65 in the control (Supplementary Figure S9A). The CRM maintained lower cut root numbers than the control, with a significant difference detected in YRL38 at 27 DAT (Supplementary Figure S9B).

#### Effect of Crown Root Number Manipulation on Shoot and Root Growth Combined With Root Cutting Treatment

**FIGURE 7:**
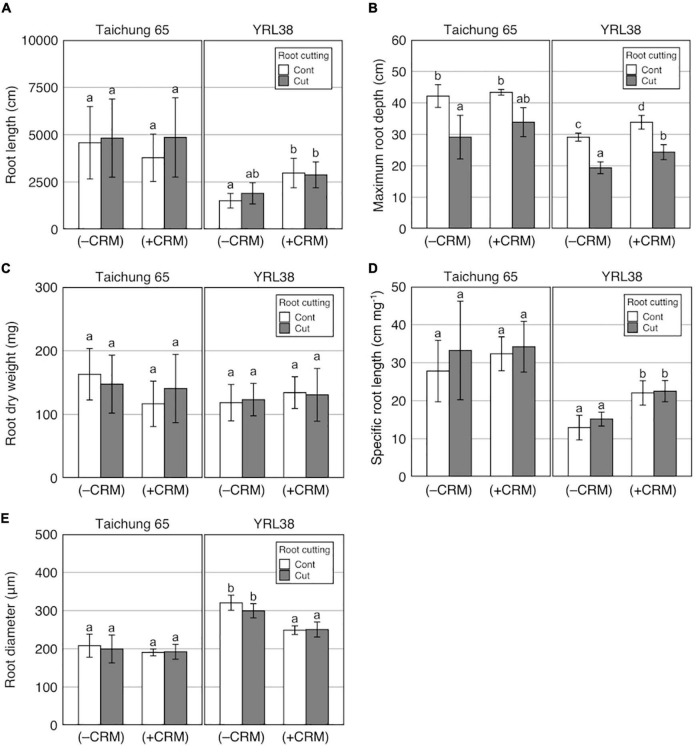
Effect of CR number manipulation (CRM) on **(A)** TRL, **(B)** MRD, **(C)** RDW, **(D)** SRL, and **(E)** RD combined with root cutting in Taichung 65 and YRL38 grown in a semihydroponic phenotyping platform for 41 days after transplanting. Values represent mean ± SD (*n* = 3–6). For each trait, bar data followed by different letters indicate significant differences among treatments in each genotype (*p* < 0.05).

Based on a three-way ANOVA, phenotypic variations between with and without CRM were detected for MRD, SCRN, RD, SRL, and LN (Table 6 and Supplementary Table S8). Genotype × CRM interactions were detected for eight traits (Table 6). TRL with CRM was higher when compared to the control without both CRM and root cutting in YRL38 but not in Taichung 65 (Figure 7A). The CRM increased MRD in YRL38 with and without root cutting (Figure 7B), also detected during early growth (Supplementary Figure S10). Among the four sections, CRM increased RL_s3 and RL_s4 of YRL38 without root cutting (Supplementary Figure S11A). In YRL38, CRM also increased RLP_s4 without root cutting and RLP_s2 with root cutting with decreased RLP_s1 (Supplementary Figure S11B). RDW did not differ among treatments with CRM for either genotype (Figure 7C), which increases SRL and decreases RD in YRL38 (Figures 7D,E). The CRM increased RL_M and RL_L in YRL38 without root cutting and RL_L with root cutting (Supplementary Figure S12A). RLP_CR decreased with CRM with and without root cutting in YRL38 (Supplementary Figure S12B). Among shoot traits, CRM increased LN in YRL38 without root cutting treatment (Supplementary Figure S13B).

**TABLE 6:** Effect of crown root number manipulation (CRM) and root cutting on 17 major traits in Taichung 65 and YRL38 grown in a semihydroponic phenotyping platform for 41 days after transplanting.

Trait	Abbreviation	Genotype	Cutting	CRM	Genotype × Cutting	Genotype × CRM	Cutting × CRM	Genotype × Cutting × CRM
Maximum root depth	MRD	***	***	**	n.s.	n.s.	n.s.	n.s.
Seminal and crown root number	SCRN	n.s.	n.s.	*	n.s.	**	n.s.	n.s.
Total root length	TRL	***	n.s.	n.s.	n.s.	n.s.	n.s.	n.s.
Root diameter	RD	***	n.s.	***	n.s.	**	n.s.	n.s.
Root area	RA	**	n.s.	n.s.	n.s.	n.s.	n.s.	n.s.
Root volume	RV	n.s.	n.s.	n.s.	n.s.	n.s.	n.s.	n.s.
Specific root length	SRL	***	n.s.	*	n.s.	n.s.	n.s.	n.s.
Root length intensity	RLI	**	***	n.s.	n.s.	*	n.s.	n.s.
Root tissue density	RTD	**	n.s.	n.s.	n.s.	n.s.	n.s.	n.s.
Root dry weight	RDW	n.s.	n.s.	n.s.	n.s.	n.s.	n.s.	n.s.
Shoot dry weight	SDW	n.s.	n.s.	n.s.	n.s.	*	n.s.	n.s.
Total dry weight	TDW	n.s.	n.s.	n.s.	n.s.	*	n.s.	n.s.
Shoot to root ratio	SRR	n.s.	n.s.	n.s.	n.s.	n.s.	n.s.	n.s.
Shoot height	SH	***	n.s.	n.s.	n.s.	n.s.	n.s.	n.s.
Leaf number	LN	n.s.	n.s.	*	n.s.	*	n.s.	n.s.
Leaf area	LA	n.s.	n.s.	n.s.	n.s.	*	n.s.	n.s.
Tiller number	TN	n.s.	n.s.	n.s.	n.s.	*	n.s.	n.s.

Significance was based on three-way ANOVA (n = 3–6) (*p < 0.05; **p < 0.01; ***p < 0.001; n.s., not significant).

## Discussion

The phenotyping study (Experiment I) identified large variation in shoot and root traits among the 20 rice genotypes. Of the 15 measured traits, 11 traits were highly varied among genotypes, with CVs > 0.20 and *p* < 0.05 (Table 3), and well correlated within and between shoot and root traits (Supplementary Table S2). For example, shoot traits such as SH and SDW had significant positive correlations with TRL (Supplementary Table S2), consistent with other studies using the semihydroponic systems in various plant species (Chen et al., 2012, 2016, 2017, 2020; Qiao et al., 2019) and soil-grown maize under drought stress (Avramova et al., 2016). Several important root traits varied among genotypes, which includes those related to root growth (TRL, SCRN, and RDW), root distribution (MRD), and economic aspects of the root system (SRL). The PCA of 13 selected major traits separated the Australian genotypes, Japanese genotypes, and mutants mainly by traits describing plant size (PC1), such as TRL, SH, and SDW, with a few exceptions including the *crl1* mutant (Figures 2A,B). In contrast, SCRN, SRL, and RD varied more within genotypes of the same group (Figures 2A,B). Many Australian rice varieties originated from the United States, where some Japanese genotypes have been introduced (Ko et al., 1994; Garland et al., 1999). Whereas Japanese and Australian varieties are closely related, the rice genotypes used in this study had significant phenotypic variations, which are probably related to the extended breeding and selection of rice genotypes adapted to the Mediterranean environments in Australia (Bajwa and Chauhan, 2017). The large phenotypic variations observed allowed us to select the genotypes with contrasting RSA for analyzing relationships between RSA and compensatory root growth.

In Experiment II, the root cutting treatment dramatically changed RSA in rice, which decreases rooting depth but maintaining TRL in the six genotypes (Figures 3C,E). None of the shoot traits was affected by root cutting (Table 5 and Supplementary Table S3), which is likely due to root growth maintenance after root cutting. Studies have shown different responses in shoot and root biomass and SRR after root cutting in lettuce (Biddington and Dearman, 1984), wheat (Vysotskaya et al., 2001; Fang et al., 2010; Ma et al., 2010), and soybean (Fanello et al., 2020). The responses depend on the root cutting methodology (e.g., ratio of removed roots, growth stage, experimental conditions). However, root cutting on a smaller portion of roots, such as just the main root tip as excised in this study, had less effect on shoot growth than root cutting on a larger portion in lettuce seedlings (Biddington and Dearman, 1984).

Distribution of compensatory roots differed among genotypes. RLs of Nipponbare, YRL38, and *qhb/Oswox5* increased in a shallow section but decreased in a deeper section, relative to the control (Figure 5). In contrast, Taichung 65 and *crl1* maintained RL at each depth (Figure 5). The proportion of compensatory RL in each section to total compensatory RL also differed among genotypes (Figure 6B). The proportion of compensatory RL in section 3 positively correlated with MRD in the control (Figure 6C) and between the control and the root cutting treatment for RL in each section (Supplementary Table S7). Therefore, deep-rooting genotypes in the control allocated more roots to deeper layers even after root cutting than shallow-rooting genotypes. Studies suggested that the RSA ideotype differs in soil environments, especially those with heterogeneous water and nutrient distribution (Schneider and Lynch, 2020; Uga, 2021). In upland conditions, deeper rooting is beneficial to drought tolerance by accessing water and nutrients from deeper soils (Uga et al., 2013). In contrast, shallow-rooted systems can efficiently acquire immobile nutrients below the soil surface (Sun et al., 2018). Drought stress severely affects crop productivity in compacted soils as the hard soil layer inhibits deep root growth (Araki and Iijima, 2005; Colombi et al., 2018). In this context, genotypes that can maintain root growth deeper in the profile would be more adaptable to compacted soils under drought through LR proliferation below the hard soil layer. Narrow pores and cracks exist in compacted soils (White and Kirkegaard, 2010; Haling et al., 2011; Kautz et al., 2013; Han et al., 2015), which provides low resistance pathways for compensatory LR growth. In contrast, in compacted soils, genotypes with a higher proportion of compensatory roots in shallow layers after root cutting might acquire water and nutrients above the hard soil layer. Therefore, selecting genotypes with compensatory root distribution at depth might increase crop production in compacted soils with varying water availability in the soil profile. Further studies are needed to test whether the observed genotypic differences in root distribution are found in compacted soils and confer shoot growth improvement under specific soil environments.

The root cutting treatment also affected root distribution in diameter classes. Most of the Australian and Japanese genotypes increased the proportion of RD length of 300–500 μm (RLP_LL) (Supplementary Figure S6B), generally below the root cutting depth (sections 2 and 3) (Figure 4C). These data indicate that root cutting induced L-type LRs which were thicker than those in the control. The increased LR diameter might be beneficial for penetrating compacted soils, as thicker roots have increased root growth pressure and potential growth rate (Materechera et al., 1992; Pagès et al., 2010), and thus greater ability to explore compacted soils. Because L-type LRs can produce higher-order LRs (Sasaki et al., 1981) and second-order LRs were observed on L-type LRs in this study (Supplementary Figure S1), the increased large L-type LR length contributed to the thinner RLs after root cutting especially below the cutting depth. *qhb/Oswox5* greatly reduced the lengths of thinner roots (<150 μm) and their proportions (Supplementary Table S6), which had more than half of TRL (Figure 4B), reducing TRL than the parent Taichung 65 (Figure 3E and Supplementary Table S3). In addition, the length of thicker roots (>150 μm) increased with root cutting, but thinner RL did not change in section 1 in the mutant (Supplementary Figure S7). *qhb/Oswox5* is defective in S-type LR formation, which includes second-order LRs, but L-type LR formation after root cutting was promoted more than in the wild-type, Taichung 65 (Kawai et al., 2022). Thus, root cutting promoted L-type LR formation in the mutant, but did not increase S-type LRs, which increases RD after root cutting (Figure 3D). The root cutting induced thick L-type LRs, which compensates root growth by producing higher-order branches.

In Experiment III, CRM promoted compensatory root growth in an Australian genotype, YRL38. The increased TRL in YRL38 was attributed to increase efficiency of root system development as reflected in the increased SRL (Figure 7D). A negative correlation between SCRN and SRL existed among the 20 rice genotypes without root tip excision (Supplementary Figure S3H), where SRL positively correlated with TRL (Supplementary Figure S3B). The CRM reduced the RLP_CR (Supplementary Figure S12B) and increased RL_L in YRL38 regardless of root cutting treatment (Supplementary Figure S12A). It is noteworthy that CRM did not decrease RL_CR in Taichung 65 and YRL38 (Supplementary Figure S12A), likely due to the promoted CR emergence in CRM during later growth (Supplementary Figure S9A). The increased SRL was also detected in *crl1* than the parent cultivar Taichung 65, recorded the highest SRL among the six genotypes (Supplementary Table S3). *crl1* has a defect in CR formation (Inukai et al., 2005) and did not produce any CRs in this study (Supplementary Table S3). Interestingly, *crl1* decreased CR length but showed similar LR growth than the parent cultivar Taichung 65 (Supplementary Figure S6 and Supplementary Table S6). Therefore, defected CR formation promoted LR growth in *crl1*. CRM also promoted deep rooting in YRL38 regardless of root cutting treatment (Figure 7B and Supplementary Figures S10, S11). These results are consistent with studies in maize, where genotypes with fewer CRs or CRM resulted in deep root systems with increased LR growth (Saengwilai et al., 2014; Gao and Lynch, 2016; Guo and York, 2019). In Taichung 65, however, MRD did not increase even with the *crl1* mutation, which did not form CRs (Figure 3C and Supplementary Table S3). CRM did not affect any root traits in Taichung 65 (Figure 7). Therefore, the effect of reduced CR number on root growth is dependent on genotype. YRL38 had a thicker root system with thicker RD of the total root system and main roots and the lowest SRL among the six tested genotypes (Figure 3D, Supplementary Figure S14, and Supplementary Table S3) and higher RLP_CR than Taichung 65 (Supplementary Table S6). Thicker roots require more assimilates for their formation and maintenance, which could reduce the assimilate allocation to LR growth (Eissenstat, 1992; Thaler and Pagès, 1999). Thus, reducing CR number might be beneficial for improving root development efficiency in genotypes with thicker root systems. Further studies are needed to identify the CR number for maximizing compensatory root growth and examine the relationship between main RD and LR development or compensatory root growth using more genotypes with contrasting main RD.

## Conclusion

The phenotyping experiment detected large variations in root and shoot traits among the 20 tested genotypes, which allows the selection of six rice genotypes with contrasting RSA. Root cutting causes compensatory root growth in rice genotypes, which did not affect TRL or RDW but altered root distribution along vertical sections and in diameter classes. After root cutting, more roots distributed at a shallower depth and thicker L-type LRs emerged, which contributes to the compensatory growth. Genotypic differences in the distribution of compensatory roots were detected—deeper rooting genotypes without root cutting allocated more compensatory roots to deeper sections than genotypes with shallower roots. The reduced CR number increased TRL with deeper root growth regardless of root cutting in an Australian genotype YRL38. The increased TRL was attributed to increased SRL, as RDW did not change. Thus, reducing CR number might be beneficial for promoting root development by improving the efficiency of root system development, but the effect will depend on genotype. The promoted compensatory LR growth by CR number manipulation might confer higher root exploration ability in compacted soils and hence better access to soil resources below the hardpan.

## Data Availability Statement

The original contributions presented in the study are included in the article/Supplementary Material, further inquiries can be directed to the corresponding author/s.

## Author Contributions

All authors conceived and designed the experiments. TK performed the experiments along the supervisions of YC, HT, YI, and KS. TK and YC analyzed the data. TK wrote the manuscript. YC, HT, YI, and KS revised the manuscript.

## Conflict of Interest

The authors declare that the research was conducted in the absence of any commercial or financial relationships that could be construed as a potential conflict of interest.

## Publisher’s Note

All claims expressed in this article are solely those of the authors and do not necessarily represent those of their affiliated organizations, or those of the publisher, the editors and the reviewers. Any product that may be evaluated in this article, or claim that may be made by its manufacturer, is not guaranteed or endorsed by the publisher.

## Abbreviations

ANOVA: analysis of variance
CR: crown root
CRM: crown root number manipulation
CV: coefficients of variation
DAT: days after transplanting
LR: lateral root
PCA: principal component analysis
P: probability
R2: R-squared (the coefficient of determination)
RSA: root system architecture
SD: standard deviation
SR: seminal root.
