# Supplementary material for: Rice Genotypes Express Compensatory Root Growth With Altered Root Distributions in Response to Root Cutting
Source: Front Plant Sci. 2022 Feb 28;13:830577. doi: 10.3389/fpls.2022.830577 (PMC8919052; doi:10.3389/fpls.2022.830577)
Supplement: Supplementary file 1 [file Data_Sheet_1.docx]

Supplementary Material

**
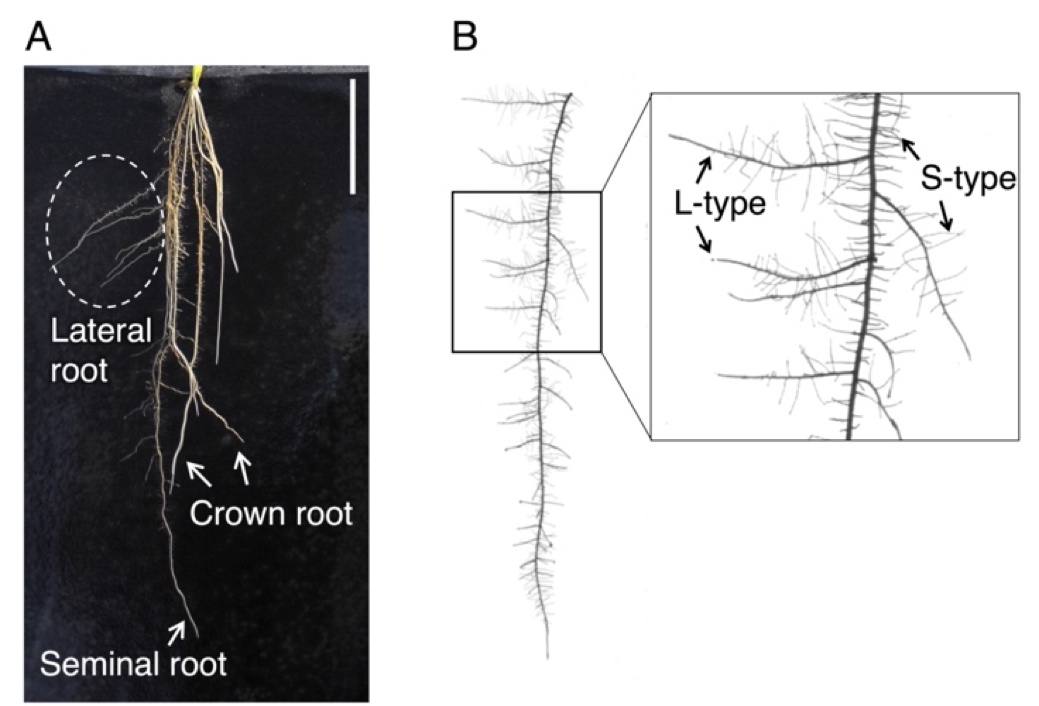
**

**Figure S1 |** Root system architecture in rice. **(A)** Root system of rice cultivar Nipponbare grown in a semi-hydroponic phenotyping platform for 30 days after transplanting. Scale bars = 5 cm. **(B)** Scanned image of crown root showing L-type and S-type lateral roots.

**
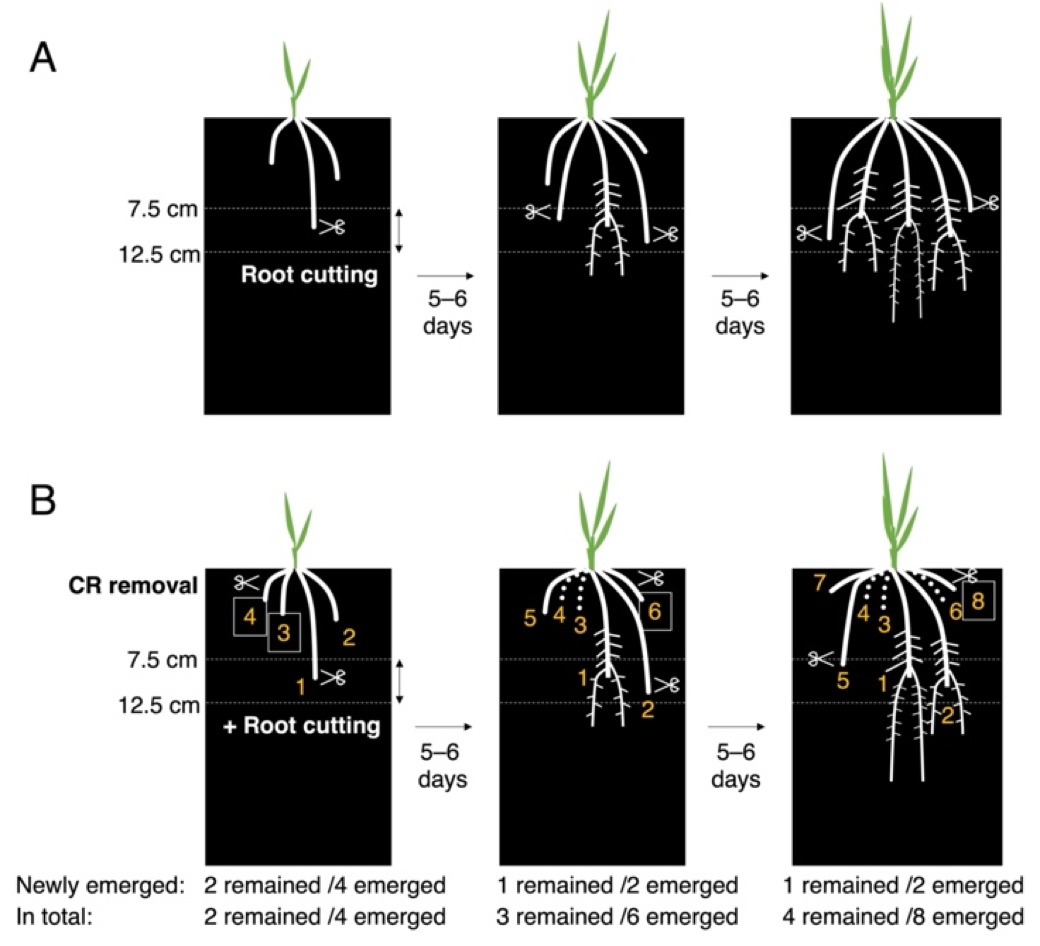
**

**Figure S2 |** Schematic diagram of **(A)** root cutting and **(B)** crown root number manipulation (CRM) combined with root cutting.

**
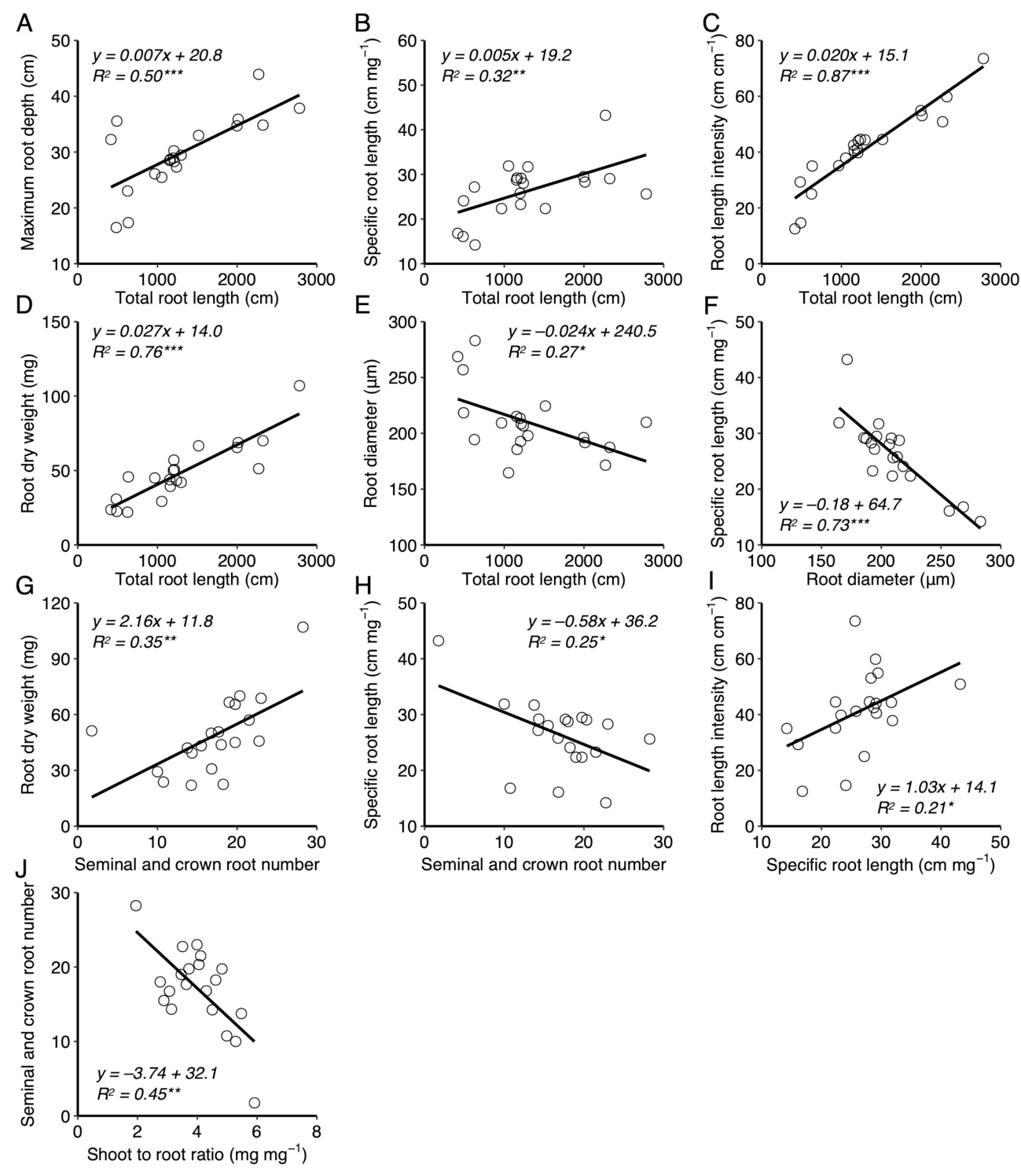
**

**Figure S3 |** Correlations between shoot and root traits in 20 rice genotypes grown in a semi-hydroponic phenotyping system for 55 days after transplanting (*, *P* < 0.05; **, *P* < 0.01; ***, *P* < 0.001).

**
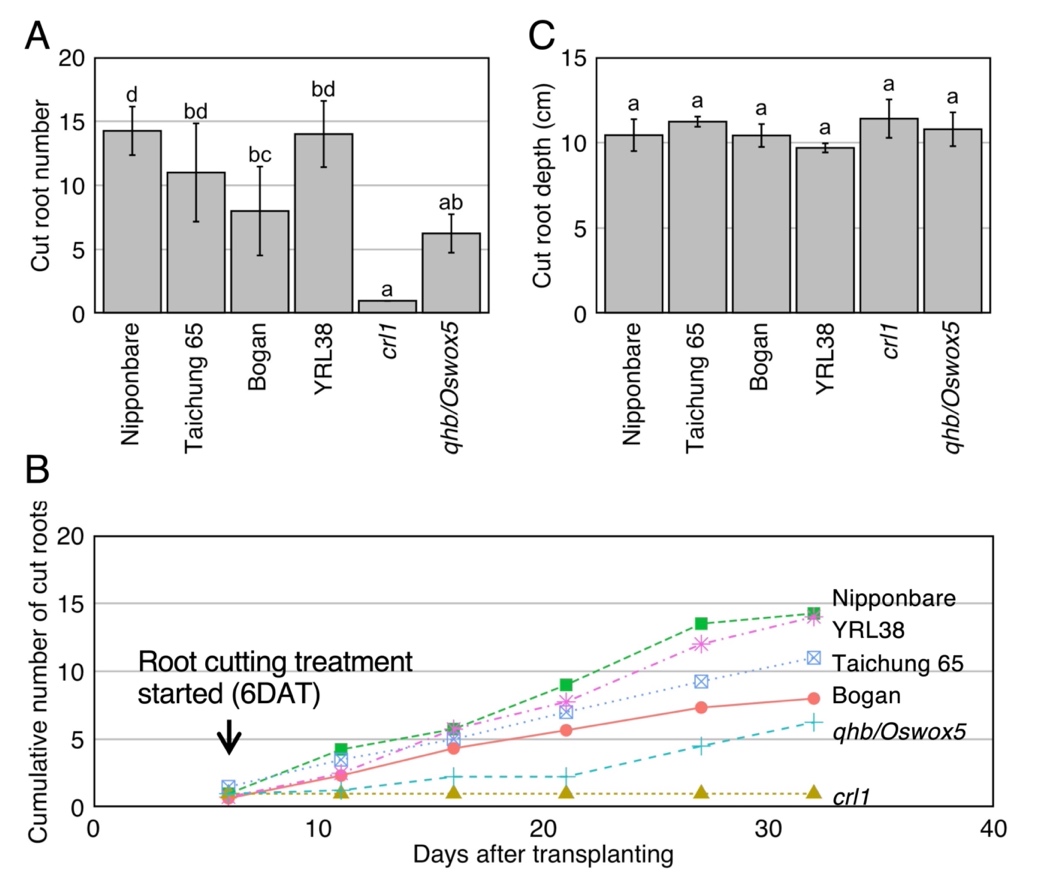
**

**Figure S4 |** Root cutting treatment in six rice genotypes grown in a semi-hydroponic phenotyping platform. **(A)** Total number of cut roots. **(B)** Cumulative number of cut roots. **(C)** Average root cutting depth. Values represent mean ± SD (*n* = 3 or 4). In **A** and **C**, bar data followed by different letters indicate significant differences among genotypes (*P* < 0.05).

**
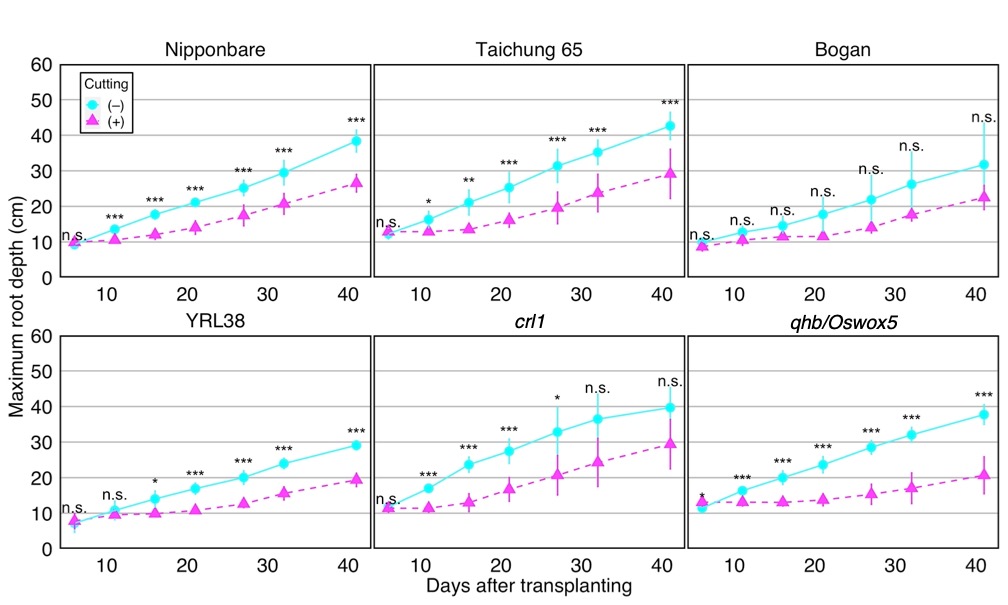
**

**Figure S5 |** Time-course analysis for the effect of root cutting on maximum root depth in six rice genotypes grown in a semi-hydroponic phenotyping platform for 41 days after transplanting. Values represent mean ± SD (*n* = 3–5) (*, *P* < 0.05; **, *P* < 0.01; ***, *P* < 0.001; n.s., not significant).

**
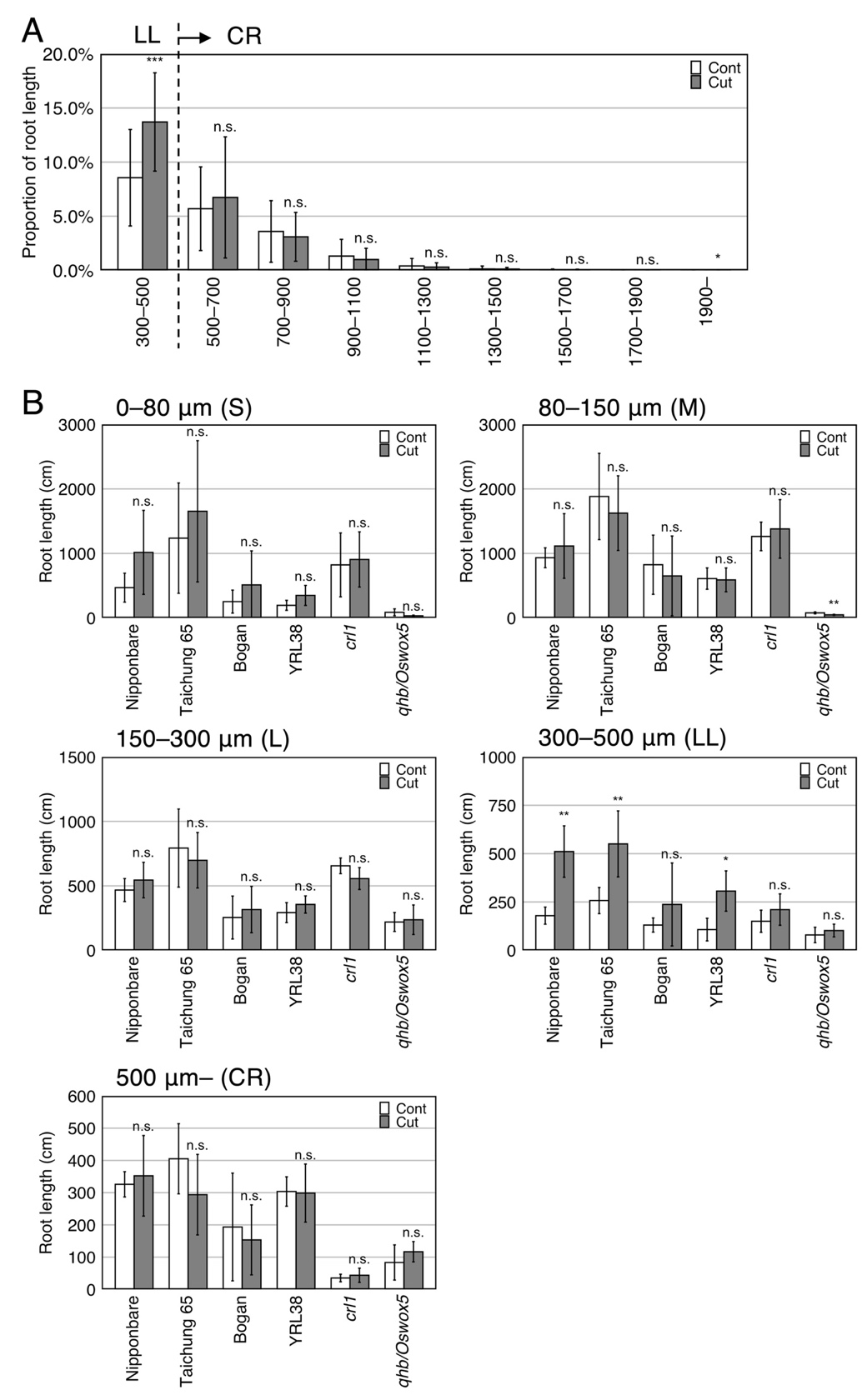
**

**Figure S6 |** Effect of root cutting treatment on root distribution in diameter classes in six rice genotypes grown in a semi-hydroponic phenotyping platform for 41 days after transplanting. **(A)** Distribution of root length proportion in diameter classes >300 μm. **(B)** Root length in five diameter classes. Values represent mean ± SD (*n* = 3–6) (*, *P* < 0.05; **, *P* < 0.01; ***, *P* < 0.001; n.s., not significant).

**
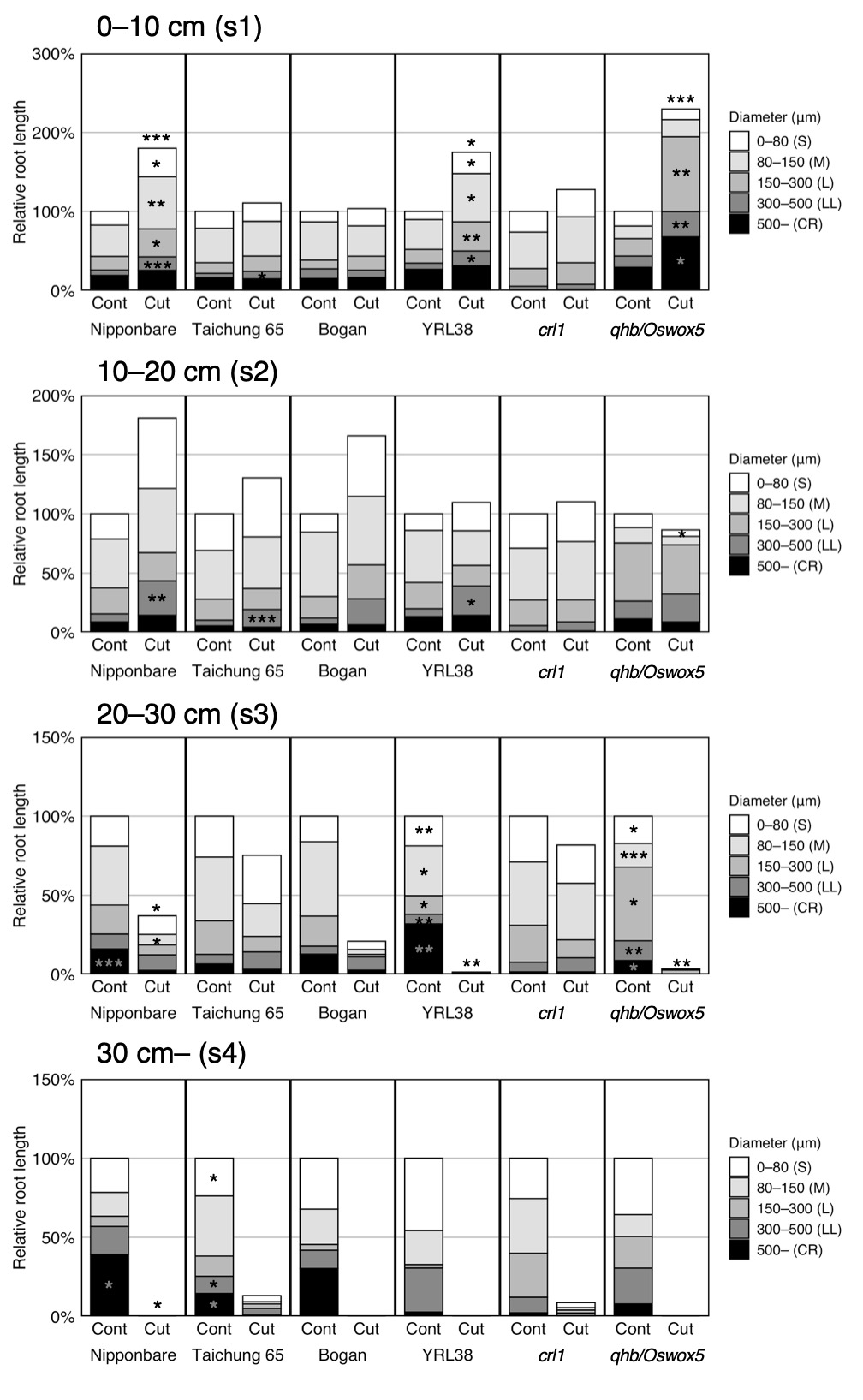
**

**Figure S7 |** Effect of root cutting treatment on root distribution in diameter classes in each section and rice genotype grown in a semi-hydroponic phenotyping platform for 41 days after transplanting. The data are expressed as relative values, with total root length in the control being 100 percent for each section of the same genotype. Values represent mean (*n* = 3–6) (*, *P* < 0.05; **, *P* < 0.01; ***, *P* < 0.001).

**
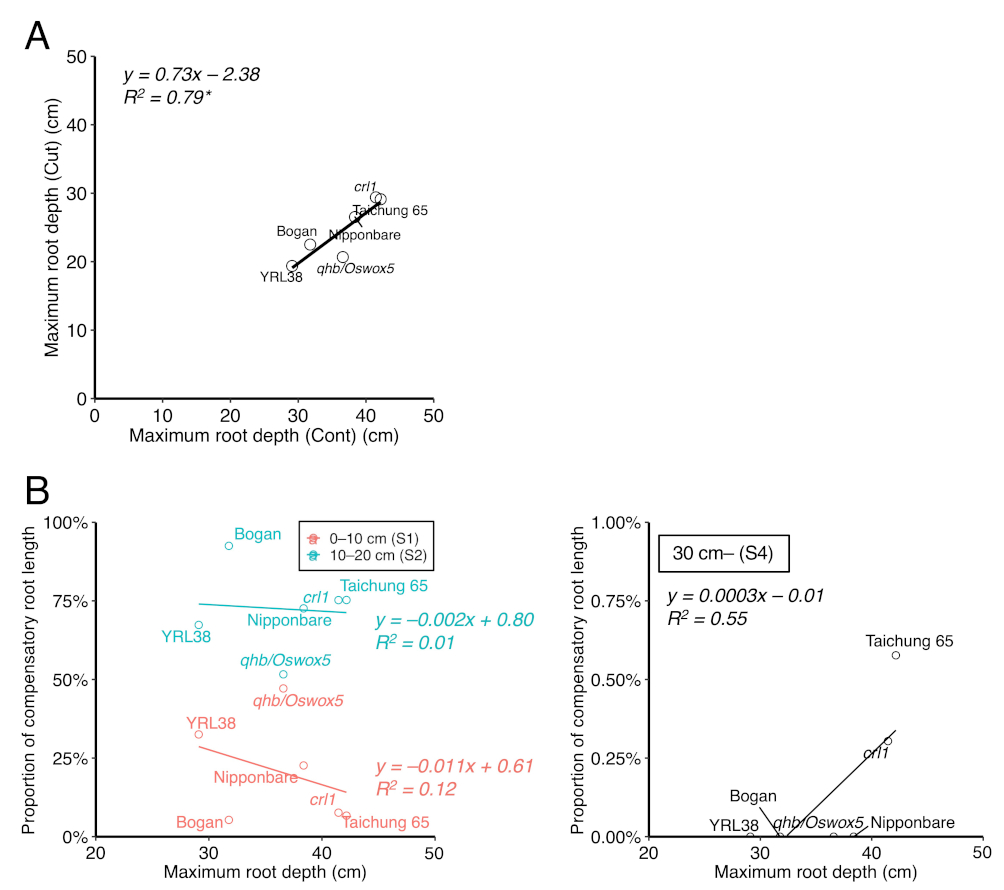
**

**Figure S8 |** Relationship between root distribution at depth in the control and root cutting treatment across six rice genotypes grown in a semi-hydroponic phenotyping platform for 41 days after transplanting. **(A)** Correlation between maximum root depth with and without root cutting (*, *P* < 0.05). **(B)** Correlations between maximum root depth in the control and proportion of compensatory root length in sections 1, 2, and 4.

**
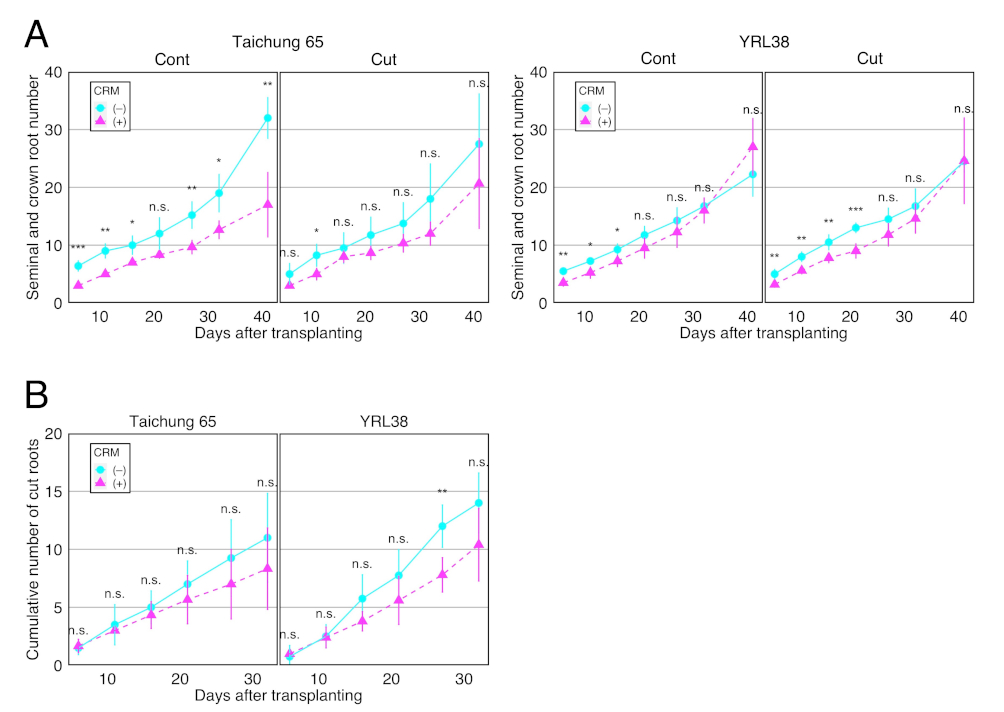
**

**Figure S9 |** Time-course analysis of the effect of crown root number manipulation (CRM) on **(A)** seminal and crown root number and **(B)** cumulative number of cut roots in Taichung 65 and YRL38 grown in a semi-hydroponic phenotyping platform. Values represent mean ± SD (*n* = 3–5) (*, *P* < 0.05; **, *P* < 0.01; ***, *P* < 0.001; n.s., not significant).

**
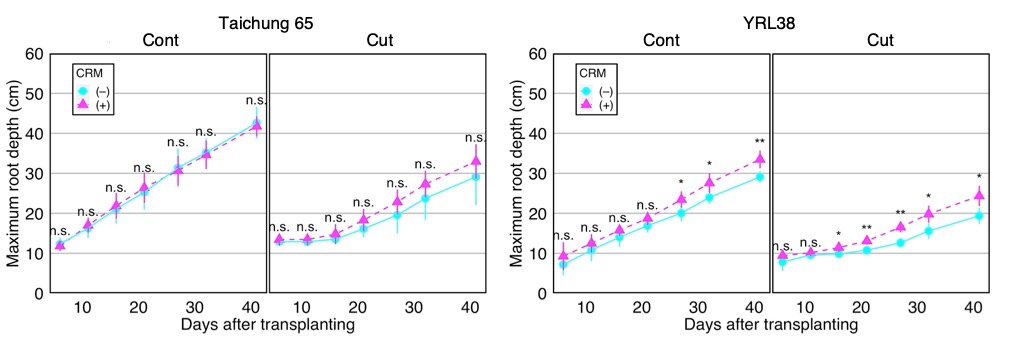
**

**Figure S10 |** Time-course analysis of the effect of crown root number manipulation (CRM) on maximum root depth in Taichung 65 and YRL38 grown in a semi-hydroponic phenotyping platform. Values represent mean ± SD (*n* = 3–5) (*, *P* < 0.05; **, *P* < 0.01; ***, *P* < 0.001; n.s., not significant).

**
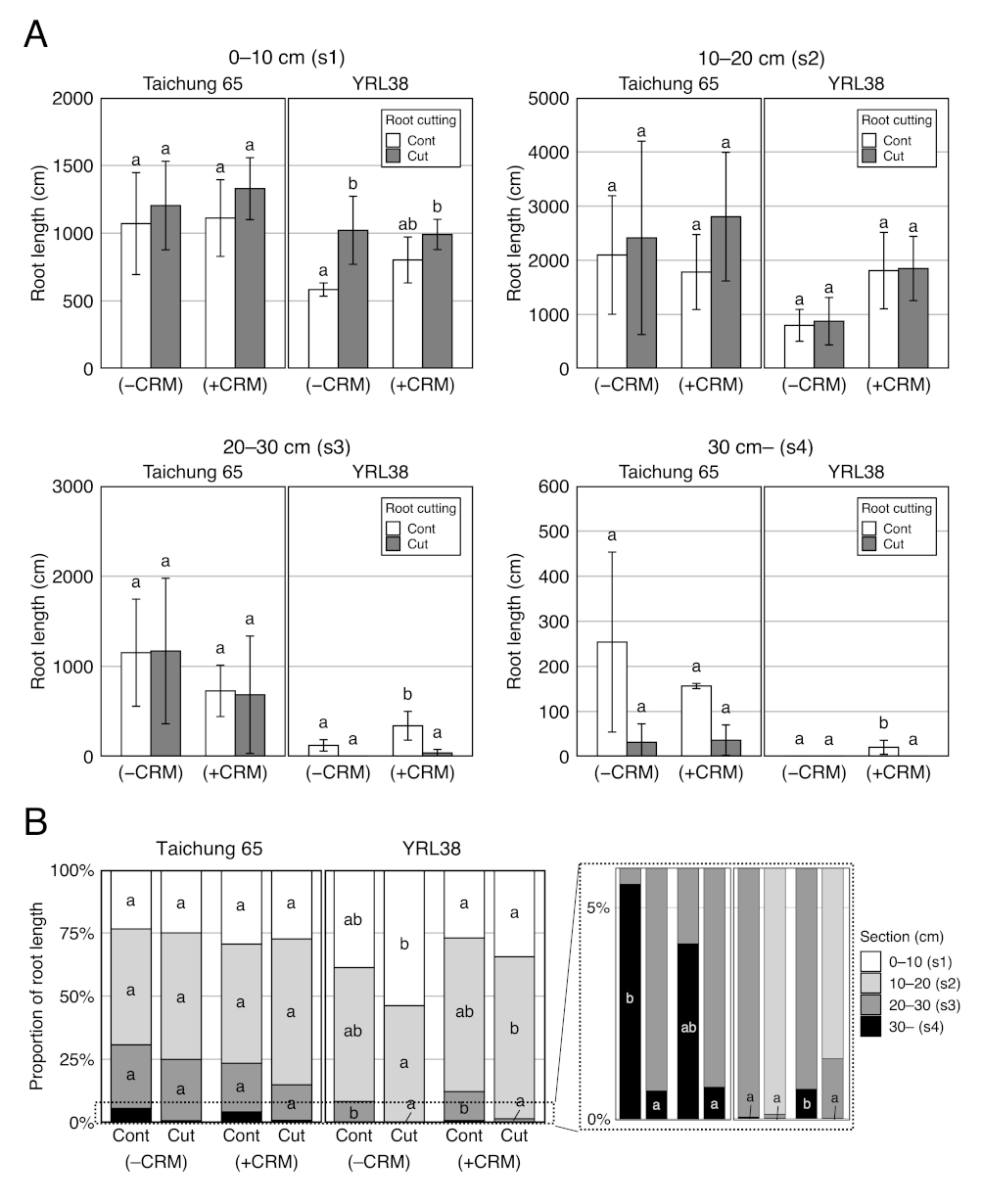
**

**Figure S11 |** Effect of crown root number manipulation (CRM) on root distribution in depth combined with root cutting in Taichung 65 and YRL38 grown in a semi-hydroponic phenotyping platform for 41 days after transplanting. **(A)** Root length in each section. **(B)** Proportion of root length in four sections. Values represent mean ± SD (*n* = 3–6). For each trait, bar data followed by different letters indicate significant differences among treatments in each genotype (*P* < 0.05).

**
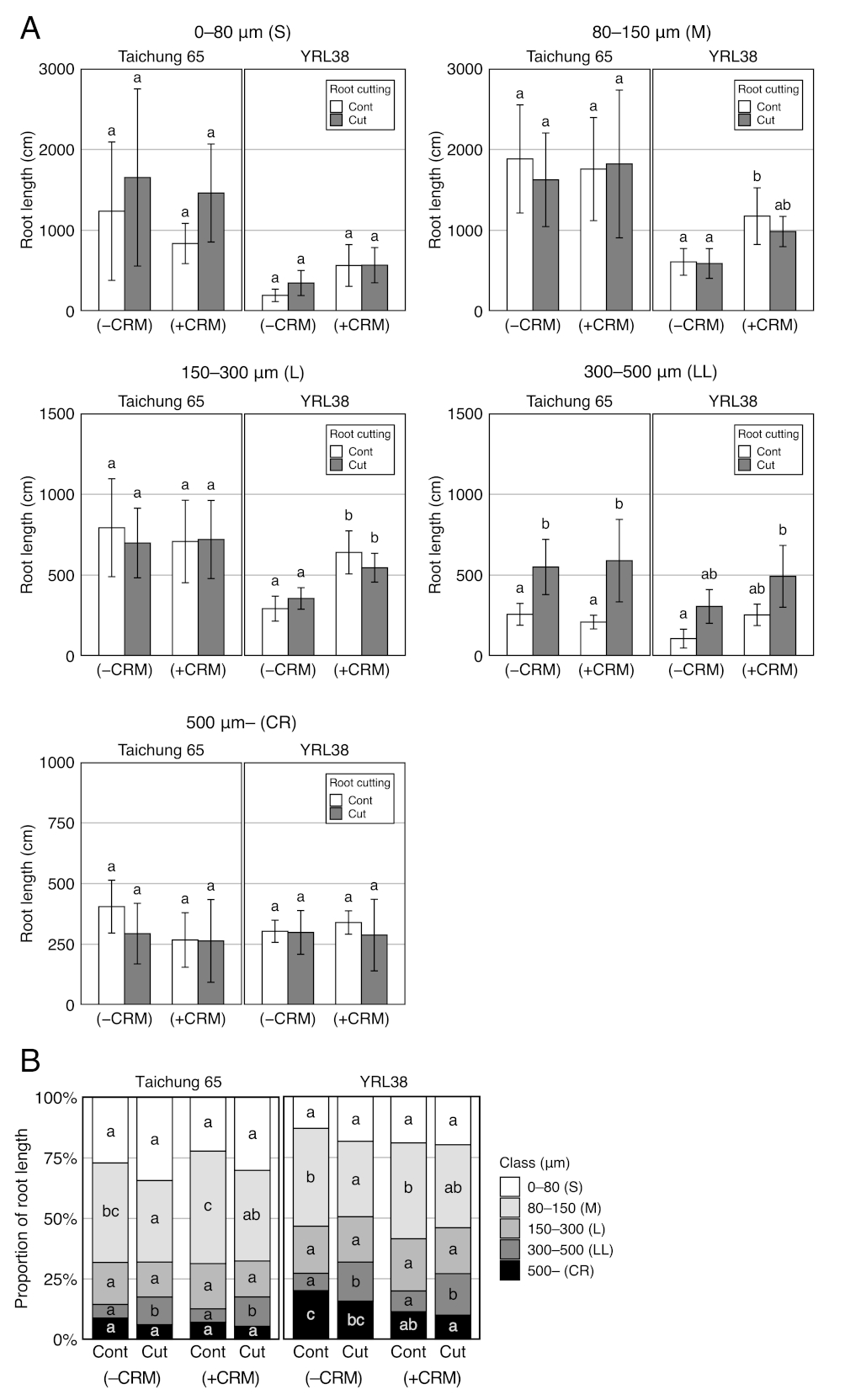
**

**Figure S12 |** Effect of crown root number manipulation (CRM) on root distribution in diameter classes combined with root cutting in Taichung 65 and YRL38 grown in a semi-hydroponic phenotyping platform for 41 days after transplanting. **(A)** Root length in each diameter class. **(B)** Proportion of root length in five diameter classes. Values represent mean ± SD (*n* = 3–6). For each trait, bar data followed by different letters indicate significant differences among treatments in each genotype (*P* < 0.05).

**
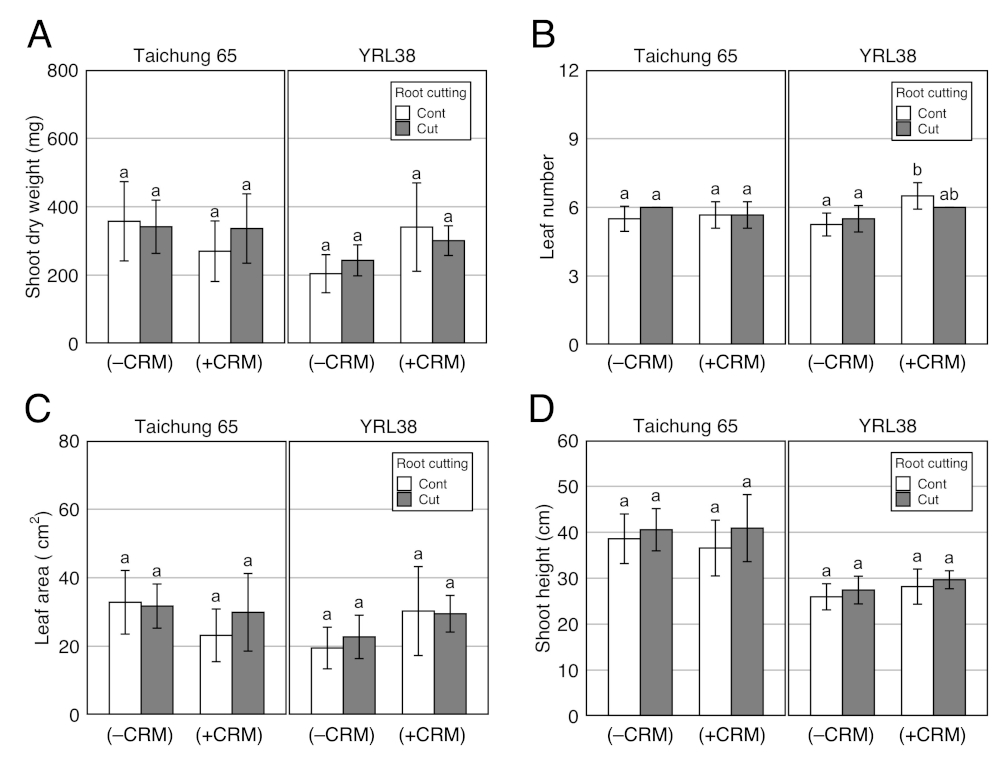
**

**Figure S13 |** Effect of crown root number manipulation (CRM) on shoot traits combined with root cutting in Taichung 65 and YRL38 grown in a semi-hydroponic phenotyping platform for 41 days after transplanting. Values represent mean ± SD (*n* = 3–6). For each trait, bar data followed by different letters indicate significant differences among treatments in each genotype (*P* < 0.05).

**
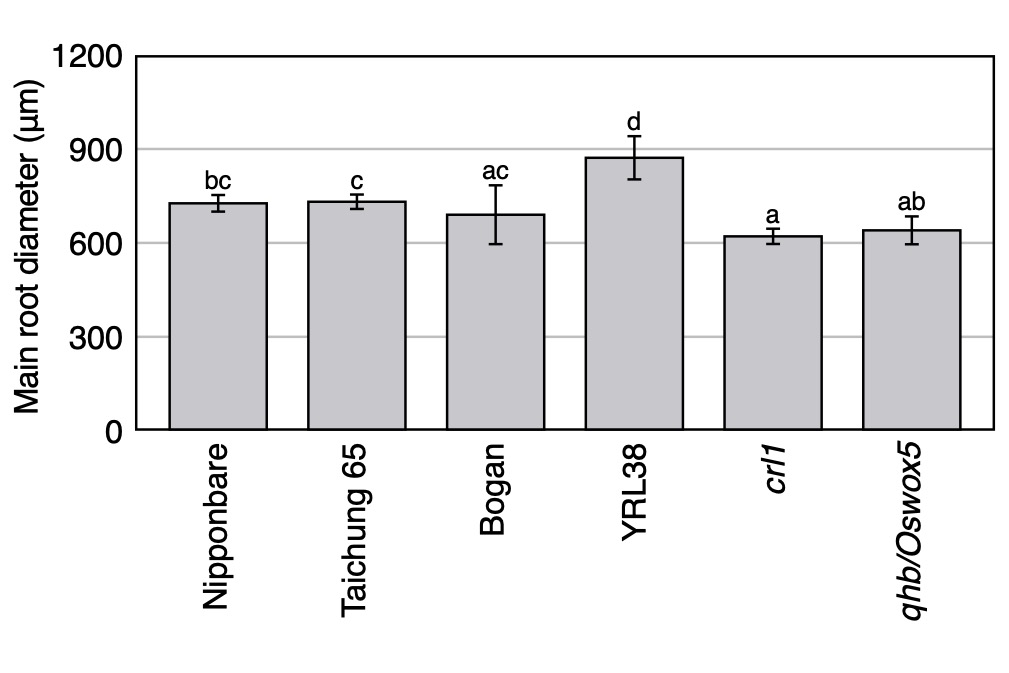
**

**Figure S14 |** Crown root diameter in six rice genotypes without root cutting treatment grown in a semi-hydroponic phenotyping platform for 41 days after transplanting. Values represent mean ± SD (*n* = 3–6). Bar data followed by different letters indicate significant differences among genotypes (*P* < 0.05).

**Table S1 |** Composition of nutrient solution. pH adjusted to 6.5 with potassium hydroxide (KOH) (Colmer 2003).

| Compound | Formula | Final concentration |
| --- | --- | --- |
| Calcium sulphate dihydrate | CaSO_4_・2H_2_O | 1.5 mM |
| MES buffer | C_6_H_13_NO_4_S・x H_2_O | 2.5 mM |
| Potassium nitrate | KNO_3_ | 3.75 mM |
| Ethylenediaminetetraacetic acid ferric sodium salt | C_10_H_12_FeN_2_NaO_8_ | 50 μM |
| Sodium metasilicate pentahydrate | Na_2_SiO_3_・5H_2_O | 0.1 mM |
| Magnesium sulphate heptahydrate | MgSO_4_・7H_2_O | 0.4 mM |
| Ammonium nitrate | NH_4_NO_3_ | 0.625 mM |
| Potassium dihydrogen phosphate | KH_2_PO_4_ | 0.2 mM |
| Potassium chloride | KCl | 50 μM |
| Boric acid | H_3_BO_3_ | 25 μM |
| Manganese sulphate monohydrate | MnSO_4_・H_2_O | 2 μM |
| Zinc sulphate heptahydrate | ZnSO_4_・7H_2_O | 2 μM |
| Copper sulphate pentahydrate | CuSO_4_・5H_2_O | 0.5 μM |
| Sodium molybdate dihydrate | Na_2_MoO_4_・2H_2_O | 0.5 μM |
| Nickel sulphate hexahydrate | NiSO_4_・6H_2_O | 1 μM |

**Table S2 |** Pearson’s correlation matrix for 15 shoot and root traits (referred to as major traits) in 20 rice genotypes grown in a semi-hydroponic phenotyping system for 55 days after transplanting. Significant correlation coefficients are in bold with asterisks (*, *P* < 0.05; **, *P* < 0.01).

|  | MRD | SCRN | RL | RD | RA | RV | SRL | RLI | RTD | RDW | SDW | TDW | SRR | SH |
| --- | --- | --- | --- | --- | --- | --- | --- | --- | --- | --- | --- | --- | --- | --- |
| SCRN | –0.12 | – | – | – | – | – | – | – | – | – | – | – | – | – |
| RL | **0.71**** | 0.22 | – | – | – | – | – | – | – | – | – | – | – | – |
| RD | –**0.48*** | 0.26 | –**0.52*** | – | – | – | – | – | – | – | – | – | – | – |
| RA | **0.65**** | 0.35 | **0.98**** | –0.37 | – | – | – | – | – | – | – | – | – | – |
| RV | **0.54*** | **0.49*** | **0.89**** | –0.15 | **0.97**** | – | – | – | – | – | – | – | – | – |
| SRL | **0.60**** | –**0.50*** | **0.57**** | –**0.86**** | 0.43 | 0.22 | – | – | – | – | – | – | – | – |
| RLI | 0.44 | 0.36 | **0.93**** | –**0.45*** | **0.95**** | **0.90**** | **0.46*** | – | – | – | – | – | – | – |
| RTD | –0.18 | 0.08 | –0.03 | –0.40 | –0.13 | –0.24 | –0.03 | –0.01 | – | – | – | – | – | – |
| RDW | **0.49*** | **0.59**** | **0.87**** | –0.19 | **0.94**** | **0.97**** | 0.15 | **0.90**** | –0.05 | – | – | – | – | – |
| SDW | **0.61**** | 0.03 | **0.80**** | –0.41 | **0.74**** | **0.63**** | **0.48*** | **0.70**** | 0.05 | **0.65**** | – | – | – | – |
| TDW | **0.63**** | 0.18 | **0.88**** | –0.38 | **0.84**** | **0.76**** | 0.43 | **0.80**** | 0.03 | **0.78**** | **0.98**** | – | – | – |
| SRR | 0.15 | –**0.67**** | –0.16 | –0.21 | –0.31 | –**0.46*** | 0.32 | –0.35 | 0.17 | –**0.47*** | 0.31 | 0.13 | – | – |
| SH | **0.56*** | 0.05 | **0.81**** | **–0.50*** | **0.75**** | **0.61**** | **0.51*** | **0.75**** | 0.06 | **0.63**** | **0.82**** | **0.83**** | 0.03 | – |
| LN | 0.41 | 0.12 | **0.49*** | 0.11 | **0.54*** | **0.56**** | 0.10 | 0.44 | –0.28 | **0.52*** | **0.68**** | **0.69**** | 0.23 | 0.38 |

**Table S3 |** Means of major traits in six rice genotypes with and without root cutting treatment grown in a semi-hydroponic phenotyping platform for 41 days after transplanting. For each trait, mean data followed by different letters indicate significant differences among genotypes (*P* < 0.05). Asterisks indicate significant differences between treatments (*n* = 3–6) (**, *P* < 0.01; ***, *P* < 0.001; n.s., not significant).

|  |  | Maximum root depth | | Seminal and crown root number | | Total root length | | Root diameter | | Root area | | Root volume | | Specific root length | | Root length intensity | | Root tissue density | |
| --- | --- | --- | --- | --- | --- | --- | --- | --- | --- | --- | --- | --- | --- | --- | --- | --- | --- | --- | --- |
|  |  | MRD (cm) | | SCRN | | RL (cm) | | RD (μm) | | RA (cm^2^) | | RV (cm^3^) | | SRL  (cm mg^–1^) | | RLI  (cm cm^–1^) | | RTD  (mg cm^–3^) | |
| Genotype | |  |  |  |  |  |  |  |  |  |  |  |  |  |  |  |  |  |  |
|  | Nipponbare | 32.5 | ab | 22.3 | c | 2957 | b | 242 | b | 218.4 | cd | 1.32 | b | 21.5 | ab | 96.5 | bc | 104.6 | a |
|  | Taichung 65 | 37.0 | b | 30.5 | d | 4673 | c | 205 | ab | 287.6 | d | 1.47 | b | 30.0 | b | 128.2 | c | 106.7 | a |
|  | Bogan | 27.1 | ab | 21.8 | c | 1764 | ab | 228 | b | 121.8 | ab | 0.71 | a | 26.2 | ab | 63.1 | ab | 100.3 | a |
|  | YRL38 | 24.2 | a | 23.4 | c | 1704 | ab | 310 | c | 164.0 | bc | 1.28 | b | 14.0 | a | 74.5 | b | 95.4 | a |
|  | *crl1* | 36.1 | b | 1.0 | a | 3003 | b | 156 | a | 144.8 | bc | 0.56 | a | 58.7 | c | 85.6 | bc | 91.5 | a |
|  | *qhb/Oswox5* | 30.2 | ab | 12.7 | b | 544 | a | 309 | c | 53.6 | a | 0.44 | a | 14.6 | a | 19.6 | a | 99.3 | a |
| Treatment | |  |  |  |  |  |  |  |  |  |  |  |  |  |  |  |  |  |  |
|  | Control | 37.3 |  | 18.4 |  | 2353 |  | 237 |  | 157.6 |  | 0.91 |  | 27.5 |  | 60.4 |  | 101.5 |  |
|  | Cut | 24.7 |  | 18.4 |  | 2660 |  | 247 |  | 177.9 |  | 1.02 |  | 28.0 |  | 100.2 |  | 97.5 |  |
|  | Significance | *** |  | n.s. |  | n.s. |  | n.s. |  | n.s. |  | n.s. |  | n.s. |  | ** |  | n.s. |  |

**Table S3 |** Continued.

|  |  | Root dry weight | | Shoot dry weight | | Total dry weight | | Shoot to root ratio | | Shoot height | | Leaf number | | Leaf area | | Tiller number | |
| --- | --- | --- | --- | --- | --- | --- | --- | --- | --- | --- | --- | --- | --- | --- | --- | --- | --- |
|  |  | RDW (mg) | | SDW (mg) | | TDW (mg) | | SRR  (mg mg^–1^) | | SH (cm) | | LN | | LA (cm^2^) | | TN | |
| Genotype | |  |  |  |  |  |  |  |  |  |  |  |  |  |  |  |  |
|  | Nipponbare | 134.9 | b | 257 | cd | 392 | bc | 1.91 | a | 32.3 | bc | 6.00 | a | 22.8 | bc | 0.75 | a |
|  | Taichung 65 | 156.8 | b | 351 | d | 508 | c | 2.28 | a | 39.4 | d | 5.70 | a | 32.4 | d | 2.00 | b |
|  | Bogan | 66.8 | a | 134 | ab | 201 | a | 1.98 | a | 21.8 | a | 5.33 | a | 13.5 | ab | 0.83 | ab |
|  | YRL38 | 120.9 | b | 224 | bc | 345 | b | 1.86 | a | 26.7 | ab | 5.38 | a | 21.3 | bc | 1.38 | ab |
|  | *crl1* | 51.1 | a | 334 | d | 385 | b | 6.57 | b | 35.1 | cd | 5.67 | a | 24.6 | cd | 1.33 | ab |
|  | *qhb/Oswox5* | 41.8 | a | 115 | a | 157 | a | 3.35 | a | 22.2 | a | 5.40 | a | 9.3 | a | 1.20 | ab |
| Treatment | |  |  |  |  |  |  |  |  |  |  |  |  |  |  |  |  |
|  | Control | 93.6 |  | 236.9 |  | 330 |  | 3.26 |  | 29.6 |  | 5.61 |  | 20.2 |  | 1.3 |  |
|  | Cut | 98.8 |  | 247.3 |  | 346 |  | 2.88 |  | 30.7 |  | 5.57 |  | 22.0 |  | 1.3 |  |
|  | Significance | n.s. |  | n.s. |  | n.s. |  | n.s. |  | n.s. |  | n.s. |  | n.s. |  | n.s. |  |

**Table S4 |** ANOVA of four shoot and root traits of six rice genotypes with and without root cutting treatment grown in a semi-hydroponic phenotyping platform for 41 days after transplanting. Significance was based on two-way ANOVA (*n* = 3–5) (*, *P* < 0.05; **, *P* < 0.01; ***, *P* < 0.001; n.s., not significant).

|  |  | Significance | | |
| --- | --- | --- | --- | --- |
|  |  | Genotype | Treatment | G×T |
| Maximum root depth (MRD) | |  |  |  |
|  | 6 DAT | *** | n.s. | n.s. |
|  | 11 DAT | *** | *** | n.s. |
|  | 16 DAT | *** | *** | * |
|  | 21 DAT | *** | *** | n.s. |
|  | 27 DAT | *** | *** | n.s. |
|  | 32 DAT | *** | *** | n.s. |
| Shoot height (SH) | |  |  |  |
|  | 6 DAT | *** | n.s. | n.s. |
|  | 11 DAT | *** | n.s. | n.s. |
|  | 16 DAT | *** | n.s. | n.s. |
|  | 21 DAT | *** | n.s. | n.s. |
|  | 27 DAT | *** | n.s. | n.s. |
|  | 32 DAT | *** | n.s. | n.s. |
| Leaf number (LN) | |  |  |  |
|  | 6 DAT | n.s. | n.s. | n.s. |
|  | 11 DAT | n.s. | n.s. | n.s. |
|  | 16 DAT | * | n.s. | n.s. |
|  | 21 DAT | n.s. | n.s. | n.s. |
|  | 27 DAT | n.s. | n.s. | n.s. |
|  | 32 DAT | n.s. | n.s. | n.s. |
| Tiller number (TN) | |  |  |  |
|  | 6 DAT | n.s. | n.s. | n.s. |
|  | 11 DAT | n.s. | n.s. | n.s. |
|  | 16 DAT | n.s. | n.s. | n.s. |
|  | 21 DAT | ** | n.s. | n.s. |
|  | 27 DAT | ** | n.s. | n.s. |
|  | 32 DAT | ** | n.s. | n.s. |

**Table S5 |** Descriptive statistics of 20 local traits in six rice genotypes with and without root cutting treatment grown in a semi-hydroponic phenotyping platform for 41 days after transplanting. Significance was based on two-way ANOVA (*n* = 3–6) (*, *P* < 0.05; **, *P* < 0.01; ***, *P* < 0.001; n.s., not significant).

| Trait | | Abbreviation | Minimum | Maximum | Mean | Median | SD | CV | Significance | | |
| --- | --- | --- | --- | --- | --- | --- | --- | --- | --- | --- | --- |
|  |  |  |  |  |  |  |  |  | Genotype | Treatment | G×T |
| Root length in sections (cm) | | | | | | | | |  |  |  |
|  | Section 1 | RL_s1 | 139.6 | 1187.0 | 757.4 | 705.6 | 332.3 | 0.44 | *** | *** | n.s. |
|  | Section 2 | RL_s2 | 212.6 | 2733.9 | 1288.0 | 1214.8 | 786.1 | 0.61 | *** | n.s. | n.s. |
|  | Section 3 | RL_s3 | 1.9 | 1151.7 | 356.6 | 228.8 | 361.3 | 1.01 | *** | n.s. | n.s. |
|  | Section 4 | RL_s4 | 0.0 | 253.9 | 44.4 | 17.9 | 73.2 | 1.65 | *** | ** | n.s. |
|  | Sections 2–4 | RL_s2–4 | 217.5 | 3633.5 | 1689.0 | 1498.1 | 1112.2 | 0.66 | *** | n.s. | n.s. |
| Root length proportion in sections | | | | | | | | |  |  |  |
|  | Section 1 | RLP_s1 | 25.6% | 60.9% | 38.0% | 35.9% | 12.1% | 0.32 | * | ** | n.s. |
|  | Section 2 | RLP_s2 | 36.7% | 58.5% | 48.5% | 50.5% | 6.7% | 0.14 | n.s. | n.s. | n.s. |
|  | Section 3 | RLP_s3 | 0.1% | 24.8% | 11.8% | 13.3% | 8.8% | 0.75 | *** | *** | n.s. |
|  | Section 4 | RLP_s4 | 0.0% | 5.2% | 1.6% | 0.4% | 2.0% | 1.25 | * | *** | n.s. |
|  | Sections 2–4 | RLP_s2–4 | 39.1% | 74.4% | 62.0% | 64.1% | 12.1% | 0.20 | * | ** | n.s. |

| Trait | | Abbreviation | Minimum | Maximum | Mean | Median | SD | CV | Significance | | |
| --- | --- | --- | --- | --- | --- | --- | --- | --- | --- | --- | --- |
|  |  |  |  |  |  |  |  |  | Genotype | Treatment | G×T |
| Root diameter length (cm) | | | | | | | | |  |  |  |
|  | D. < 80 μm | RL_S | 32.7 | 1653.1 | 627.6 | 490.7 | 502.6 | 0.80 | *** | n.s. | n.s. |
|  | 80 ≤ D. < 150 μm | RL_M | 48.3 | 1882.7 | 916.8 | 879.0 | 567.8 | 0.62 | *** | n.s. | n.s. |
|  | 150 ≤ D. < 300 μm | RL_L | 220.0 | 793.1 | 449.5 | 411.8 | 197.7 | 0.44 | *** | n.s. | n.s. |
|  | 300 ≤ D. < 500 μm | RL_LL | 79.8 | 550.4 | 235.3 | 195.2 | 153.9 | 0.65 | *** | *** | ** |
|  | D. ≥ 500 μm | RL_CR | 35.3 | 405.0 | 217.1 | 243.7 | 128.3 | 0.59 | *** | n.s. | n.s. |
| Root diameter length proportion | | | | | | | | |  |  |  |
|  | D. < 80 μm | RLP_S | 6.4% | 31.3% | 20.5% | 21.5% | 7.3% | 0.36 | *** | n.s. | n.s. |
|  | 80 ≤ D. < 150 μm | RLP_M | 9.2% | 49.6% | 34.6% | 37.1% | 11.9% | 0.34 | *** | *** | *** |
|  | 150 ≤ D. < 300 μm | RLP_L | 14.2% | 42.6% | 22.4% | 19.5% | 9.2% | 0.41 | *** | n.s. | n.s. |
|  | 300 ≤ D. < 500 μm | RLP_LL | 5.5% | 19.0% | 11.0% | 11.1% | 4.5% | 0.41 | *** | *** | n.s. |
|  | D. ≥ 500 μm | RLP_CR | 1.3% | 22.8% | 11.6% | 11.0% | 6.6% | 0.57 | *** | n.s. | * |

**Table S5 |** Continued.

**Table S6 |** Means of local traits in six rice genotypes with and without root cutting treatment grown in a semi-hydroponic phenotyping platform for 41 days after transplanting. For each trait, mean data followed by different letters indicate significant differences among genotypes (*P* < 0.05). Asterisks indicate significant differences between treatments (*n* = 3–6) (*, *P* < 0.05; **, *P* < 0.01; ***, *P* < 0.001; n.s., not significant).

|  |  | Root length in sections | | | | | | | | | | Root length proportion in sections | | | | | | | | | |
| --- | --- | --- | --- | --- | --- | --- | --- | --- | --- | --- | --- | --- | --- | --- | --- | --- | --- | --- | --- | --- | --- |
|  |  | Section 1 | | Section 2 | | Section 3 | | Section 4 | | Sections 2–4 | | Section 1 | | Section 2 | | Section 3 | | Section 4 | | Sections 2–4 | |
|  |  | RL_s1 (cm) | | RL_s2 (cm) | | RL_s3 (cm) | | RL_s4 (cm) | | RL_s2–4 (cm) | | RLP_s1 | | RLP_s2 | | RLP_s3 | | RLP_s4 | | RLP_s2–4 | |
| Genotype | |  |  |  |  |  |  |  |  |  |  |  |  |  |  |  |  |  |  |  |  |
|  | Nipponbare | 878 | bc | 1743 | bc | 305 | a | 30 | a | 2079 | b | 32.1% | a | 55.1% | a | 11.6% | ab | 1.2% | a | 67.9% | a |
|  | Taichung 65 | 1117 | c | 2352 | c | 1038 | b | 166 | b | 3556 | c | 27.0% | a | 49.1% | a | 20.6% | b | 3.3% | a | 73.0% | a |
|  | Bogan | 611 | ab | 952 | ab | 177 | a | 24 | ab | 1153 | ab | 46.6% | a | 44.7% | a | 7.6% | ab | 1.1% | a | 53.4% | a |
|  | YRL38 | 803 | bc | 838 | ab | 63 | a | 0 | a | 901 | ab | 47.9% | a | 48.0% | a | 4.0% | a | 0.0% | a | 52.1% | a |
|  | *crl1* | 881 | bc | 1541 | bc | 520 | a | 61 | ab | 2122 | b | 30.6% | a | 50.8% | a | 16.3% | ab | 2.3% | a | 69.4% | a |
|  | *qhb/Oswox5* | 212 | a | 233 | a | 84 | a | 16 | a | 332 | a | 39.7% | a | 43.1% | a | 14.6% | ab | 2.6% | a | 60.3% | a |
| Treatment | |  |  |  |  |  |  |  |  |  |  |  |  |  |  |  |  |  |  |  |  |
|  | Control | 637 |  | 1134 |  | 490 |  | 92 |  | 1716 |  | 30.6% |  | 46.9% |  | 19.1% |  | 3.3% |  | 69.4% |  |
|  | Cut | 892 |  | 1492 |  | 269 |  | 7 |  | 1769 |  | 43.7% |  | 50.3% |  | 5.8% |  | 0.1% |  | 56.3% |  |
|  | Significance | * |  | n.s. |  | n.s. |  | ** |  | n.s. |  | ** |  | n.s. |  | *** |  | *** |  | ** |  |

**Table S6 |** Continued.

|  |  | Root diameter length | | | | | | | | | | Root diameter length proportion | | | | | | | | | |
| --- | --- | --- | --- | --- | --- | --- | --- | --- | --- | --- | --- | --- | --- | --- | --- | --- | --- | --- | --- | --- | --- |
|  |  | 0–80 μm | | 80–150 μm | | 150–300 μm | | 300–500 μm | | 500 μm+ | | 0–80 μm | | 80–150 μm | | 150–300 μm | | 300–500 μm | | 500 μm+ | |
|  |  | RL_S (cm) | | RL_M (cm) | | RL_L (cm) | | RL_LL (cm) | | RL_CR (cm) | | RLP_S | | RLP_M | | RLP_L | | RLP_LL | | RLP_CR | |
| Genotype | |  |  |  |  |  |  |  |  |  |  |  |  |  |  |  |  |  |  |  |  |
|  | Nipponbare | 743 | ac | 1024 | bc | 506 | bc | 345 | bc | 339 | d | 22.4% | ab | 35.5% | b | 18.3% | a | 11.5% | ab | 12.3% | bc |
|  | Taichung 65 | 1403 | c | 1780 | d | 755 | d | 375 | c | 361 | d | 27.2% | b | 39.1% | bc | 16.7% | a | 8.4% | a | 8.5% | b |
|  | Bogan | 383 | ab | 738 | bc | 286 | ab | 184 | ac | 174 | bc | 19.3% | ab | 41.5% | bc | 17.2% | a | 11.1% | ab | 10.8% | bc |
|  | YRL38 | 272 | ab | 600 | ab | 324 | ab | 207 | ac | 301 | cd | 15.0% | a | 35.6% | b | 19.5% | a | 11.4% | ab | 18.5% | c |
|  | *crl1* | 860 | bc | 1315 | cd | 612 | cd | 177 | ab | 39 | a | 27.1% | b | 44.1% | c | 21.5% | a | 6.0% | a | 1.3% | a |
|  | *qhb/Oswox5* | 65 | a | 66 | a | 227 | a | 89 | a | 97 | ab | 12.3% | a | 12.9% | a | 41.2% | b | 16.1% | b | 17.4% | c |
| Treatment | |  |  |  |  |  |  |  |  |  |  |  |  |  |  |  |  |  |  |  |  |
|  | Control | 552 |  | 955 |  | 470 |  | 154 |  | 222 |  | 19.5% |  | 36.8% |  | 23.7% |  | 8.5% |  | 11.5% |  |
|  | Cut | 755 |  | 912 |  | 458 |  | 323 |  | 212 |  | 22.1% |  | 30.6% |  | 22.4% |  | 13.6% |  | 11.3% |  |
|  | Significance | n.s. |  | n.s. |  | n.s. |  | *** |  | n.s. |  | n.s. |  | n.s. |  | n.s. |  | *** |  | n.s. |  |

**Table S7 |** Pearson’s correlations among root length in sections between the control and root cutting treatment across six genotypes grown in a semi-hydroponic phenotyping platform for 41 days after transplanting (*, *P* < 0.05; **, *P* < 0.01; ***, *P* < 0.001).

|  |  | Root length (Control) | | | | |
| --- | --- | --- | --- | --- | --- | --- |
|  |  | Section 1 | Section 2 | Section 3 | Section 4 | Sections 2–4 |
| Root length (Cut) | Section 1 (0–10 cm) | 0.84* | 0.84* | 0.62 | 0.51 | 0.75 |
|  | Section 2 (10–20 cm) | 0.88* | 0.93** | 0.87* | 0.79 | 0.92* |
|  | Section 3 (20–30 cm) | 0.85* | 0.93** | 0.98*** | 0.98*** | 0.97** |
|  | Section 4 (>30 cm) | 0.78 | 0.85* | 0.95** | 0.98*** | 0.91* |
|  | Sections 2–4 (>10 cm) | 0.91* | 0.97** | 0.94** | 0.88* | 0.97** |

**Table S8 |** Descriptive statistics for 17 shoot and root traits (referred to as major traits) in Taichung 65 and YRL38 with and without root cutting combined with and without crown root number manipulation grown in a semi-hydroponic phenotyping platform for 41 days after transplanting (*n* = 3–6).

| Trait | Abbreviation | Minimum | Maximum | Mean | Median | SD | CV |
| --- | --- | --- | --- | --- | --- | --- | --- |
| Maximum root depth (cm) | MRD | 19.4 | 43.4 | 31.9 | 31.5 | 8.2 | 0.26 |
| Seminal and crown root number | SCRN | 17.0 | 32.5 | 24.5 | 24.6 | 4.7 | 0.19 |
| Total root length (cm) | RL | 1509 | 4856 | 3412 | 3377 | 1306 | 0.38 |
| Root diameter (μm) | RD | 190 | 320 | 239 | 228 | 50 | 0.21 |
| Root area (cm^2^) | RA | 150.8 | 293.3 | 235.6 | 230.5 | 52.8 | 0.22 |
| Root volume (cm^3^) | RV | 1.10 | 1.51 | 1.37 | 1.44 | 0.15 | 0.11 |
| Specific root length (cm mg^–1^) | SRL | 12.9 | 34.2 | 25.0 | 25.2 | 8.2 | 0.33 |
| Root length intensity (cm cm^–1^) | RLI | 51.9 | 160.2 | 106.4 | 102.0 | 33.8 | 0.32 |
| Root tissue density (mg cm^–3^) | RTD | 89.8 | 109.8 | 99.4 | 99.2 | 7.5 | 0.08 |
| Root dry weight (mg) | RDW | 117 | 163 | 134 | 133 | 16 | 0.12 |
| Shoot dry weight (mg) | SDW | 204 | 358 | 299 | 319 | 55 | 0.18 |
| Total dry weight (mg) | TDW | 323 | 521 | 434 | 453 | 69 | 0.16 |
| Shoot to root ratio (mg mg^–1^) | SRR | 1.7 | 2.5 | 2.2 | 2.4 | 0.3 | 0.12 |
| Shoot height (cm) | SH | 26.0 | 40.9 | 33.5 | 33.1 | 6.3 | 0.19 |
| Leaf number | LN | 5.3 | 6.5 | 5.8 | 5.7 | 0.4 | 0.07 |
| Leaf area (cm^2^) | LA | 19.5 | 32.8 | 27.5 | 29.7 | 4.9 | 0.18 |
| Tiller number | TN | 1.0 | 2.3 | 1.6 | 1.6 | 0.5 | 0.30 |
